# A U.S. Pharmacopeia (USP) overview of Pan American botanicals used in dietary supplements and herbal medicines

**DOI:** 10.3389/fphar.2024.1426210

**Published:** 2024-08-30

**Authors:** Roy Upton, Ignacio Agudelo, Yadira Cabrera, Armando Caceres, Angela Calderón, Fernando Calzada, Rosa Camacho, Fernando da Costa, Cecilia Dobrecky, Roberto Enciso, Marcela Escobar, Mina Fakhary, Edward Fletcher, Quanyin Gao, Olga Lock, Rachel Mata, Mirtha Parada, Wilmer Perera, Luis Miguel Pombo, Eike Reich, Eric Sanchez, Mario Juan Simirgiotis, Christian Sood, Virginie Treyvaud Amiguet, Martha Villar, Ricardo Ghelman, Mariana Cabral Schveitzer, Caio Fábio Schlechta Portella, Adriana Wolffenbüttel, Bettina Ruppelt, Fabiana Souza Frickmann, Janette Gavillan-Suarez, Kristin Allen, Luis Diego Alvarado, Nandakumara Sarma, Robin Marles, Maria Monagas, Mirtha Navarro-Hoyos

**Affiliations:** ^1^ American Herbal Pharmacopoeia, Scotts Valley, CA, United States; ^2^ Facultad de Farmacia y Bioquímica, Departamento de Farmacología, Cátedra de Farmacobotánica, Universidad de Buenos Aires, Buenos Aires, Argentina; ^3^ Ministerio de Salud Publica del Ecuador, Cuenca, Ecuador; ^4^ Laboratorios Farmaya, Ciudad de Guatemala, Guatemala; ^5^ Department of Drug Discovery and Development, Harrison College of Pharmacy, Auburn University, Auburn, AL, United States; ^6^ Unidad de Investigación Médica en Farmacología, UMAE Hospital de Especialidades 2 Piso CORSE, Centro Médico Nacional Siglo XXI, Instituto Mexicano del Seguro Social, Cuidad de Mexico, Mexico; ^7^ Direccion General de Medicamentos, Insumos y Drogas (DIGEMID), Ministerio de Salud Publica del Peru, Lima, Peru; ^8^ Faculdade de Ciencias Farmaceuticas de Ribeirao Preto, Universidad de Sao Paulo, Ribeirao Preto, Brazil; ^9^ Universidad de Buenos Aires, Facultad de Farmacia y Bioquímica, Departamento de Tecnología Farmacéutica, Cátedra de Tecnología Farmacéutica I, Buenos Aires, Argentina; ^10^ Farmacopea de los Estados Unidos Mexicanos, Cuidad de Mexico, Mexico; ^11^ Facultad de Farmacia, Universidad de Valparaiso, Valparaiso, Chile; ^12^ Pharmavite LLC, Valencia, CA, United States; ^13^ Native Botanicals Inc., Banner Elk, NC, United States; ^14^ Herbalife, Quality Control Labs, Los Angeles, CA, United States; ^15^ Federacion Latinoamericana de Asociaciones Quimicas (FLAQ), Lima, Peru; ^16^ Facultad de Quimica, Universidad Autonoma de Mexico (UNAM), Cuidad de Mexico, Mexico; ^17^ Agencia Nacional de Medicamentos (ANAMED), Instituto de Salud Publica de Chile, Gran Santiago, Chile; ^18^ CAMAG Scientific Inc., Wilmington, NC, United States; ^19^ Centro de Investigacion Fundacion Universitaria Juan N. Corpas, Bogota, Colombia; ^20^ CAMAG Laboratory, Muttenz, Switzerland; ^21^ Medical Sciences Campus, University of Puerto Rico, San Juan, PR, United States; ^22^ Instituto de Farmacia, Facultad de Ciencias, Universidad Austral de Chile, Valdivia, Chile; ^23^ The Reena Group, Mississauga, Canada; ^24^ Health Canada, Ottawa, ON, Canada; ^25^ Centro de Investigación Clínica de Medicina Complementaria (CICMEC), Gerencia de Medicina Complementaria, Seguro Social de Salud-EsSalud and Departamento de Medicina Preventiva y Salud Pública, Facultad de Medicina, Universidad Mayor de San Marcos, Lima, Peru; ^26^ Natural Products Committee of the Brazilian Academic Consortium or Integrative Health (CABSIN), San Pablo, Brazil; ^27^ TRAMIL Program, San Jose, PR, United States; ^28^ Department of Drug Discovery and Development, Harrison School of Pharmacy, Auburn University, Auburn, AL, United States; ^29^ Department of Chemistry, University of Costa Rica (UCR), Bioactivity & Sustainable Development (BIODESS) Group, San Jose, Costa Rica; ^30^ United States Pharmacopeia (USP) Botanical Dietary Supplements and Herbal Medicines Expert Committee, United States Pharmacopeia (USP), Rokcville, MD, United States; ^31^ United States Pharmacopeial Convention (USP), Dietary Supplements and Herbal Medicines, Rockville, MD, United States; ^32^ Department of Chemistry, Georgetown University, Washington, DC, United States

**Keywords:** botanicals, dietary supplements, standards, quality, herbal medicines

## Abstract

The United States Pharmacopeial Convention (USP) is a nonprofit, scientific, standard-setting organization, and world leader in establishing quality, purity, and testing standards for medicines, foods, and dietary supplements. USP quality standards are used in more than 140 countries and are legally recognized by more than 40 countries. Currently, there is renewed interest in herbal medicines globally, and health policies are being implemented worldwide for the use of complementary and traditional medicine. In response, USP has developed a robust body of monographs that can be used to guide industry and regulators in ensuring the quality and safety of botanical ingredients used in dietary supplements and herbal medicines. Throughout the Pan American regions, there is a strong tradition of using botanicals as herbal medicines and, as in other regions, a growing desire for botanical dietary supplements. This underscores the need for public quality standards to ensure quality, reduce the flow of substandard and adulterated products, and ensure public health and safety. In April 2022, USP launched the Pan America Botanical Dietary Supplements and Herbal Medicines Expert Panel, with experts representing 12 different countries. The Expert Panel’s work focuses on developing quality control standards for the most important botanical ingredients used in the respective countries, ingredients that are also of global importance. This article provides an overview of the state of botanical dietary supplements and herbal medicines in different Pan American regions with a focus on the regulatory status of herbal products, the development of national quality and research initiatives, and policies related to agriculture conservation and sustainability, among other topics.

## 1 Introduction

Pharmacopeias are officially recognized sources of quality standards for identity, strength, purity, and limits of contaminants for ingredients and preparations used as medicines. Until approximately the 1930s, the majority of medicines formally accepted in medical practice throughout most of the world were derived from botanical ingredients. These medicines comprised a mix of crude Galenical preparations (teas, pills, tinctures, syrups, etc.) and substances isolated from plants (e.g., digitalis glycosides). Even today, the majority of the global population utilizes some form of traditional healing practices for their healthcare needs, and botanicals are integral in these practices (Bodeker et al., 2005; WHO, 2019).

For the context of this paper, we refer to the terms “herbal medicines,” “traditional medicine,” and “complementary medicine”, as defined by the World Health Organization and Pan American Health Organization (WHO/PAHO). “Herbal medicines” include herbs, herbal materials, herbal preparations, and finished herbal products that contain, as active ingredients, parts of plants, other plant materials, or combinations thereof (WHO, 2010). “Traditional medicine” is the sum total of the knowledge, skill, and practices based on the theories, beliefs, and experiences indigenous to different cultures, whether explicable or not, used in the maintenance of health as well as in the prevention, diagnosis, improvement, or treatment of physical and mental illness. “Complementary medicine” and “alternative medicine” refer to a broad set of healthcare practices that are not part of that country’s own traditional or conventional medicine and are not fully integrated into the dominant healthcare system. They are used interchangeably with traditional medicine in some countries (WHO, 2010). Also important is to define the term “Traditional, complementary and integrative medicine health product” as defined in the recent. WHO, 2024: includes products of various classifications generally used as medicines or for health purposes including, but not limited to “botanical medicines”, “complementary medicines”, “dietary supplements”, “food supplements”, “health supplements”, “herbal medicinal products”, “herbal medicines”, “herbal products”, “natural health products”, or “traditional medicines”. It also includes any other health technologies and devices originating from or specific to traditional, complementary and integrative medicine (WHO, 2024).

Some herbal medicines are primarily used in self-care, and others are incorporated into formal medical practices. In addition, according to WHO/PAHO, with increased healthcare costs, herbal medicines are expected to play a role in preventing chronic diseases and meeting the needs of aging populations, leading to better recognition of the role of Traditional and Complementary Medicine (T&CM) in national health systems (WHO, 2010; WHO, 2019). In recent decades, most nations have experienced a resurgence of interest in botanical medicines. This interest is driven by a mix of new scientific studies confirming the safety and efficacy of some traditional herbal drugs, along with commercial marketing, consumer demand, and international initiatives that attempt to ensure equity and access to high-quality healthcare for the world’s population (WHO, 1978; WHO, 2018).

WHO reported that 88% of member countries acknowledge the use of herbal medicines within the context of T&CM (WHO, 2019). Herbal medicine is integral to traditional medicine practices and plays an important role in providing the primary form of healthcare to most of the world’s indigenous populations, who are often marginalized and have little access to conventional medical care. The accessibility, relatively low cost, and cultural relationship with plants are among the factors driving indigenous herbal medicine use. The ever-increasing market of botanical dietary supplements and herbal medicines to the general public is a worldwide phenomenon, primarily to complement conventional therapies and improve the accessibility of all therapeutic options (WHO, 2019).

The quality assessment of botanical drugs and dietary supplement ingredients is highly complex because botanical ingredients are subject to both inherent natural variation due to genetic and environmental factors and variation introduced by processing techniques. In addition, botanical ingredients are characterized by a chemical complexity that poses analytical challenges when compared to purified drug compounds. Historically, materia medica treatises, followed later by works of modern pharmacognosy, provided the basis for quality control of botanical ingredients. Great emphasis was placed on the sourcing of raw materials and ensuring their proper identification, purity, quality, and lack of adulteration, which are quality parameters still required today. As synthetic medicinal chemistry and pharmacology advanced as dominant scientific disciplines, less focus was placed on the plant itself and more attention was given to isolated or active compounds. The subsequent proliferation of botanical extracts representing only a fraction of the plant’s phytochemical components also brought many challenges, still very apparent today. Improper extract characterization before their use in clinical studies can result in inconsistencies and unclear conclusions. Also, the positive or negative results of one study on a particular botanical cannot be inherently extrapolated to other studies conducted with a different preparation of the same botanical (Oketch-Rabah et al., 2018; Núñez Sellés and Nriagu, 2019; Heinrich et al., 2022). The historically accumulated knowledge of medicinal plant quality (i.e., growing habitat, optimal harvesting and processing conditions) became increasingly difficult to find. At the same time, modern phytochemical and pharmacological investigations provide valuable tools that can further advance herbal medicine knowledge and quality.

USP was founded in 1820, specifically to provide medical communities with processes and guidance designed to ensure that medicines were made in a manner that provided a high level of confidence in their safety and efficacy based on well-defined standards (Brinckmann et al., 2020). Beginning in 1908, early editions of the *U.S. Pharmacopeia* were translated into Spanish and adopted by some national authorities, thus expanding the breadth and scope of monographs beyond U.S. borders. This practice has been facilitated by the regularly updated, online publication of a certified Spanish translation of the current *U.S. Pharmacopeia–National Formulary (USP–NF)* (USP, 2024a). Today, USP standards are included in the regulatory frameworks of more than 40 countries, and are recognized and used in over 140 countries, in many cases through specific laws and regulations (USP, 2024b). While most of the traditional botanical products in the Unites States are regulated as dietary supplements and only a few remain as recognized medicines, some Pan American nations have greater preservation of traditional medicines practices, including the use of herbal medicines, than exists in Canada, Europe, and the United States. USP has a strong commitment to the development of pharmacopeial standards for botanical drugs in the USP-NF (USP, 2024c), botanical ingredients and products regulated as dietary supplements in the United States, which are published in the Dietary Supplements section of USP–NF and the Dietary Supplements Compendium (DSC) (USP, 2024d), as well as for botanicals used as traditional herbal medicines, which are published in the Herbal Medicines Compendium (HMC) (USP, 2024e).

**FIGURE 1:**
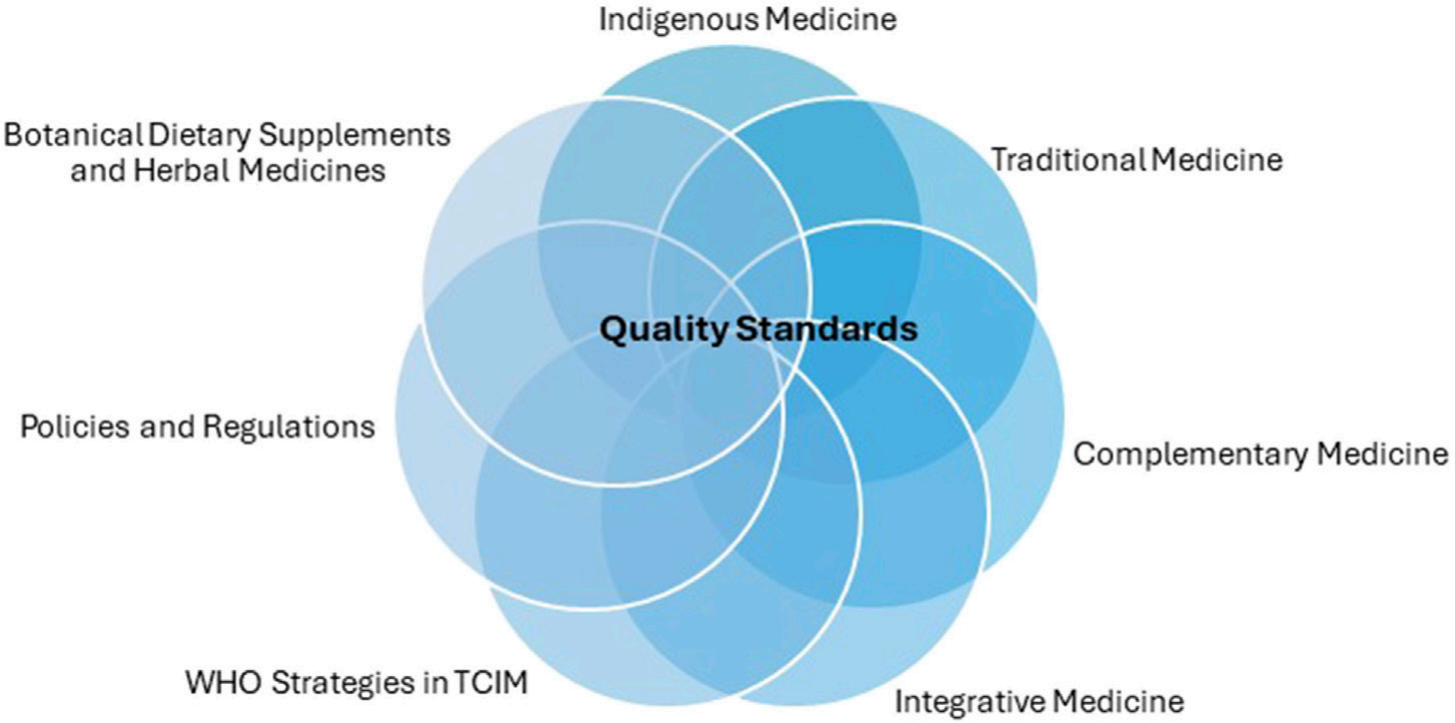
Quality Standards for Botanical Dietary Supplements and Herbal Medicines are central to fulfilling public health strategies, and implementation of policies and regulations to sustain the different medicine practices guaranteeing safety and efficacy, independent of the regulatory framework existing throughout the Pan Am region.

In addition, numerous botanicals that originate from the Pan American region are found in supplements sold in the United States. This underscores the need for quality control standards both within and beyond U.S. borders. Pan-American countries are also unique in that there is a much greater representation of indigenous healing practices, which have led to the integration of herbal medicines to greater or lesser degrees into the national primary health system in some countries of the region. These healing practices predominantly use relatively crude herbal preparations, in contrast to the finished products represented by pills, capsules, and concentrated extracts that are more representative of modern herbal preparations. In some countries from the Pan American region, including Bolivia, Brazil, Cuba, Mexico, and Peru, some herbal ingredients are considered essential medicines because they satisfy the priority healthcare needs of the population. Standards for identity, purity, strength, and limits of contaminants established by pharmacopeias are applicable to all types of articles of botanical origin and combined with conforming to good manufacturing practices provide a safety net to protect public health, and support the implementation of Traditional, Complementary and Integrative Medicine (TCIM) strategies and practices (Figure 1). However, according to WHO, the member states from the Americas region face many challenges regarding regulatory issues in the practice of T&CM, particularly concerning the lack of mechanisms to monitor the safety and overall quality of T&CM products, among other important challenges (WHO, 2019).

USP launched the Pan American Botanical Dietary Supplements and Herbal Medicines (BDSHM) Expert Panel in April 2022 under the direction of the USP Botanical DSHM Expert Committee. The Expert Panel monitors and considers the scientific, technical, and policy issues associated with monographs and candidate reference standard materials for botanical ingredients, with emphasis on articles used in the traditional systems of the Pan American region. The creation of new USP standards is intended to support the quality of botanicals used as dietary supplements and herbal medicines in the Pan American region, and these standards are made available for adoption by various pharmacopeias and stakeholders including manufacturers, laboratories, governments, and regulatory agencies.

Working with members of the Pan American BDSHM Expert Panel, the herbal landscape of each country was surveyed and summarized. The following is an overview of the historical evolution of the use of herbal medicines, the regulatory framework, and the research initiatives in Pan American regions, together with the portfolio of solutions that USP offers to address the quality of botanical dietary supplements and herbal medicines and thereby helping to improve global public health.

## 2 Historical use of Pan American plants

Throughout the Pan American region, there is a long history of herbal medicine use that began with the indigenous peoples of each nation. Due to the relative proximity and similar environmental conditions of Canada and the United States, as well as similar timelines and patterns in national development, the herbal traditions and practices are generally consistent with each other. The cultural and environmental similarities throughout Mexico and most of South and Central America similarly have resulted in herbal traditions that are more similar than different from each other, often using different species within the same genus, but there are distinct differences when compared with Canada and the Unites States. The ecosystems found within each nation contribute to their unique floral diversities. In the northern, more temperate hemisphere, numerous plant species of Canada, the Unites States., and northern Mexico overlap, while the equatorial tropical climates of South and Central America offer a greater degree of botanical diversity.

There are also similarities among the ways that various herbal traditions evolved into the herbal medicine practices and markets observed today witshin the entire Pan American region. Indigenous peoples of each country, prior to European expansion, only used medicines derived from their environment and trade with neighboring peoples; these included botanicals, animals, minerals, and ritual (Aguilar et al., 1994; Bye, 2016). Common to all these traditional healing practices, as occurs elsewhere in the world, is the predominant use of medicinal plants. The indigenous knowledge of each country was primarily transmitted orally, so written records of indigenous plant use are of post-European origin, that may not faithfully represent traditional pre-European practice. Supplementary Table S1 includes a historical timeline summarizing main milestones in the use of herbal medicines in the Pan Am region.

### 2.1 United States, Canada and Mexico

The earliest North American ethnobotanical literature originated from Mexico. Among the earliest written records is *Libellus de Medicinalibus Indorum Herbis* (*Book of Medicinal Herbs of the Indies*, also known as the *Badianus Manuscript*), published in 1552. This text was written in the Nauhuatl language of the Aztecs by Martin Cruz, a native physician (Gates, 1939); a Spanish version was published by the Economic Culture Fund and the Mexican Institute of Social Security. Another was *Rerum Medicarum Novae Hispaniae thesaurus seu plantarum, animalium, mineralium, Mexicanorum historia* (*Inventory of medical items from New Spain, or, History of Mexican plants, animals and minerals*) published by Spanish ethnographer Francisco Hernández (Hernández, 1651). Other writings were transmitted across continents in numerous languages and were later translated into English, such as *La historia medicinal de las cosas que se traen de nuestras Indias Occidentales* (*The medicinal history of the things that are brought from our West Indies*) by Spanish physician and botanist Nicholas Monardes (Monardes, 1580), translated by John Frampton as the *Joyfull Newes out of the New-Found Worlde* (Frampton, 1596). Many of the plants recorded in these earliest manuscripts (e.g., achiote (annatto) seed, capsicum pepper fruit, papaya fruit) were included in the New World materia medica, herbals, and pharmacopoeias that emerged later and they and their extracts and isolates are included in pharmacopoeial monographs worldwide today.

The first *Pharmacopoeia of the United States* (*U.S. Pharmacopeia*) was published in 1820 (Brinckmann et al., 2020). It was divided into sections, beginning with the front matter, historical introduction, and preface, followed by the materia medica (a primary list of 217 drugs, including 145 botanicals or botanically derived ingredients. This section was followed by a secondary list of 71 drugs with ingredients of “doubtful efficacy” whose use was uncertain, including 67 botanicals. From *USP I* version (1831) onward, the monographs included brief botanical descriptions with macroscopic and organoleptic properties to be verified for identification of the articles. Standards of identity, purity, and strength, and additional detailed information on each *USP* article, were included in *The Dispensatory of the United States of America*, which was published from 1833 to 1973 (Brinckmann et al., 2020). Some botanicals that first appeared in *USP* in 1820, including both native American species for use in indigenous medicine and imported species, are still currently included in *USP–NF*, USP’s official compendium, and correspond to top-selling herbs in the U.S. market today as dietary supplement ingredients, especially aloe, black cohosh, garlic, ginger, senna, and turmeric.

The *Mexican Pharmacopeia*, published in 1846, was the second in the Americas after *USP* (FEUM: Farmacopea de los Estados Unidos Mexicanos, 2024). Approximately 52.7% of the content of the first version corresponded to articles of botanical origin. In 1930, the *National Pharmacopeia* was published by the Department of Health of Mexico. Those materials whose therapeutic usefulness or applications were not validated according to certain established criteria were excluded. Unfortunately, this eliminated many of the medicinal plants that, until then, had been included in previous pharmacopeias and that were still being used in pharmacies. In 2000, the first edition of the *Herbal Pharmacopeia of the United Mexican States* was published (FEUM: Farmacopea de los Estados Unidos Mexicanos, 2024). This edition reintroduces medicinal plants, including 76 botanical monographs, and recognizes again the national materia medica as part of the official drugs within Mexico.

In Canada and the United States., in recent decades, there has been a resurgence of interest and important representation of indigenous herbal medicine practices by First Nations–Native Americans, and immigrants from different countries. In several communities, the prevalence of traditional healing practices varies, such as the widespread importance of *botanicas* to Latino immigrants in New York City (Parker, 2017) or the practice of *Curanderismo* among Latino communities in New Mexico, in which free online classes are provided (Torres, 2019).

In the United States, healthcare services are provided primarily through employer-based health insurance, Medicare, or Medicaid. Because of this, there is an entire culture of Latinos not covered due to immigration status or low-paying jobs who seek traditional healing practices “because they are accessible, affordable, and provide culturally and linguistically compatible care, indicating that they offer an alternative that addresses systemic structural barriers to biomedical healthcare” (Cruz et al., 2022). A large percentage of the herbal products used in these communities lack formal quality control standards, may not be manufactured according to good manufacturing practices, and for some, are subject to adulteration and filth (Palhares et al., 2020), creating an opportunity and need for appropriate quality control standards to be established for a wide range of botanicals and communities.

Mexico holds 10%–12% of the world’s biodiversity, ranking fourth globally in the variety of vascular plants that exist. There are approximately 31,000 different species, 3,000 to 5,000 of which form part of the medicinal flora (Aguilar et al., 1994; Aguilar-Contreras et al., 1998; Villaseñor, 2016; Rodríguez-Hernández et al., 2022). In Mexico there is such a robust trade and use of traditional and relatively crude herbs and their preparations in-home care, by local healers, and through the many herb and food markets that is often described as “one of the most impressive survivals of ancient Mexico” (Peterson, 1960). In past market surveys, 114 to 150 different plants were reportedly traded in the markets (Nicholson and Arzeni, 1993; Bye, 2016), while the number of species used by some communities is considerably higher, for example, 445 by the Zapotecs (Frei et al., 1998). Considering that up to 5,000 plants in Mexico have recorded medicinal use, the limited number of botanicals found in markets and used by individual communities likely represents those species for which there is strong ethnobotanical evidence for safety and efficacy.

Canada, Mexico, and the United States have experienced significant growth in interest in herbal products for a variety of reasons. In the United States, the 2022 retail herb market that is trackable was estimated at approximately $12.12 billion, just a slight decline from 2021 ($12.35 billion) compared to a growth of more than 17% in 2020, apparently fueled by the COVID pandemic (Smith et al., 2024). As there are untracked markets, this must be considered an underestimate. According to a U.S. survey of supplement use between 2007 and 2010, 49% of Americans said they used a supplement in the past 30 days (Bailey et al., 2013). The most commonly reported reasons for using supplements were to “improve” (45%) or “maintain” (33%) overall health. Women used calcium products for “bone health” (36%), whereas men were more likely to report supplement use for “heart health or to lower cholesterol” (18%). Older adults (≥60 years) were more likely than younger individuals to report the use of supplements for heart, bone and joint, and eye health. Most of these were used for self-care, as only 23% of products were used based on recommendations of a healthcare provider. The findings showed that supplement users, as compared with nonusers, were more physically active, were less likely to smoke, had higher educational attainment and socioeconomic status, were more likely to report very good or excellent health, have health insurance, use alcohol moderately, and exercise more frequently than nonusers. This suggests that the use of supplements is predominantly part of a general healthy lifestyle. Other reported reasons for use were for energy, mental health, prostate health, weight loss, and menopause (Bailey et al., 2013). There are scarce detailed reports of Canadian sales, but anecdotally, Canadian sales are thought to track approximately 10% of the U.S. market, with sales of similar botanicals and product categories.

In Mexico, about 80%–90% of the population reportedly utilizes some form of traditional herbal medicine and an estimated 50,000 shops sell herbal products, but the actual sales remain underestimated because a large portion of herbal sales, such as sales by traditional healers, are not tracked (FNIHMATN, 2021; Rodríguez-Hernández et al., 2022; López Obrador, 2023). In addition to consumer use, 54% of health professionals and 49% of physicians reportedly used medicinal plants as an alternative therapy for several diseases (Alonso-Castro et al., 2017). Market surveys, trends, and empirical feedback suggest that the primary uses of herbal medicines and nutritional supplements include digestive disorders, anxiety, inflammation, vascular disorders, and respiratory diseases (Nicholson and Arzeni, 1993; Aguilar-Contreras et al., 1998). The growth of the Mexican herbal market is in part fueled by the longstanding traditional integration of medicinal plants into domestic and traditional healing practices and is also due in part to the lower cost of herbal preparations compared to modern medications and treatments.

### 2.2 Central America and the Caribbean

The herbal medicine traditions of the three Virgin Islands and the Caribbean region, in general, are similar and are a blend of the knowledge of original Carib indigenous, the traditions of Spanish conquistadors, and African descents, together with the recent influx of herbal products based on American commercial popularity (Morton, 1981; Thomas et al., 1997). There are two primary populations in the Virgin Islands consisting of African-American descendants of the Caribbean slave trade and French West Indies dominated by those who migrated from more southern islands of Guadeloupe, Martinique, Saint Barthelemy, and St. Martin, and a smaller representation of “non-islanders” from a variety of areas of the Unites States and elsewhere (Yong, 2019).

The U.S. territory of Puerto Rico has a greater diversity of flora than the Virgin Islands. According to medicinal plant’s with significant use surveys, 118 plants were recorded as being used in Puerto Rico to treat depression, nervousness, chronic sinusitis, gastritis, gastroesophageal reflux disease, allergic rhinitis, rhinopharyngitis, asthma, arthritis, and migraine (Alvarado-Guzmán et al., 2009).

In the Caribbean islands and the countries associated with the Caribbean Basin including continental central, north- and south American nations, the TRAMIL (Traditional Medicines in the Islands) initiative was established in 1982 to help provide high-quality primary care to those who live within the Caribbean basin, leveraging the existing cultural use of medicinal plants, which is endemic throughout the region (TRAMIL, 2017). TRAMIL’s applied research into the use of local medicinal plants, includes an ethnopharmacological survey in conjunction with experimental *in vivo* and vitro assessments of safety and efficacy, and a review of the scientific literature regarding the botany, chemistry, toxicology, biological activities, dosage and form of preparation, and other relevant information, with an emphasis on safety and efficacy.

The purpose of this work was summed up as follows in the second edition of TRAMIL’s *Caribbean Herbal Pharmacopoeia*: “to reduce the cost of therapeutic medications, by providing grassroots communities and paramedical personnel with practical knowledge concerning the treatment of certain common ailments that may be cured by plants at a minimal cost and in harmony with popular tradition” and “to stimulate action-oriented research that has the potential to educate physicians, pharmacologists, health personnel, and those involved in primary healthcare programs” (Weniger and Robineau, 1988).

More than 48 TRAMIL ethnopharmacological surveys have been completed in territories of the Caribbean (Germosén-Robineau et al., 2017; Boulogne et al., 2011; Clement et al., 2015). TRAMIL also disseminates information regarding efficacy and toxicity studies on the use of botanical remedies to the community, primary healthcare, and governmental levels. Since the mid-1980s, more than 90 medicinal plants have been evaluated and subsequently recognized in various Caribbean nations including Cuba, the Dominican Republic, Honduras, Nicaragua, and Panamá as effective treatments that can be incorporated into primary healthcare programs (Farnsworth et al., 1985; Hoareau and DaSilva, 1999). TRAMIL’s work further describes the importance of knowing the distinction between simple belief and what is a useful and effective remedy (TRAMIL, 2017). The results of TRAMIL work are provided in the online publication of the *Caribbean Herbal Pharmacopoeia*, now in its third edition.

The primary goals of the TRAMIL initiative remain to provide low-cost access to primary and other sustainable health services in the Caribbean Basin, to better inform stakeholders regarding the rational use and scientific basis for traditional medicinal plant use, to preserve traditional knowledge and subject it to scientific review, and to establish programs that protect species from extinction. TRAMIL does not support the commercialization of herbal medicines, and its community health goals focus on providing low-cost alternatives for the treatment of self-limiting ailments either for use by health professionals or in self-care when formal medical care is not needed.

For instance, in Jamaica, a TRAMIL survey identified 107 medicinal plants from 51 plant families used to treat illnesses or maintain health (Picking et al., 2015). The use of plants was recorded as highest for mental health issues (nerves, insomnia, etc.), for respiratory problems (cold/flu/cough, etc.), and health maintenance with tonics (blood cleansers). Forty-two percent (113/270) of medicinal plant users utilized combinations of more than one plant. Leaf material was the most commonly used plant part (69%), and fresh material (98%) was most commonly prepared as a decoction for ingestion or oral use (87%). As a testimony to the integration of these plants into primary healthcare, most medicinal plants (75%) were sourced from backyards, and grandmothers (33%) and mothers (32%) were the main sources of information about plant use. Jamaicans reported limited use of other complementary and alternative medicine, underscoring the reliance on local medicinal plants for personal health (Picking et al., 2015).

Guatemala is a polyethnic country in the heart of the Mesoamerican region. Most of the country is composed of 22 different ethno-linguistical groups of Mayan origin, with smaller representation of Ch’orti’ and Afro-Caribbean groups, known as Garifunas or Caribes. The botanical and cultural richness of Guatemala has been described as “a legacy of the Maya civilization” and as reflecting a “confluence of Northern and Southern floras as well as the syncretism of Western and Eastern medical beliefs” (Girón et al., 1991). Numerous investigators have cataloged the ethnobotanical traditions of the region since the late 1920s (Mejía, 1927; Aguilar-Girón, 1958; Mellen, 1974; Dieseldorff, 1977; Cáceres et al., 1987a; Cáceres et al., 1987b; Cáceres et al., 1990; Cáceres et al., 1991; Kufer et al., 2005; Audet et al., 2012; Vargas and Andrade-Cetto, 2018; Cáceres et al., 2019; Castañeda et al., 2022; Castañeda et al., 2023). Medicinal plants include native and endemic species, but also American plants that were exchanged in pre-Colombian times. During the Spanish conquest, international medicinal plants became integrated with native species.

Generally, herbal products are well accepted by consumers, both because they require no prescription and because they belong to a culturally aligned approach to healthcare. In addition to the National Vademecum of Medicinal Plants of Guatemala, a Central American Medicinal Plants Vademecum is being developed and includes 150 medicinal plants that are traditionally used and commercially available in Central America; these include native, introduced, and imported species and products. A Pan American Compendium is also under development and consists of therapeutic monographs of 122 native species along with a priority list of 30 native plants that was prepared by a multisectoral commission [Commission of Medicinal Plants from Northern Central America (CIPLANCE)] (Cáceres, 2009; Cáceres and Cáceres, 2019).

Costa Rica represents one of the biodiversity hotspots of the world. In fact, current reports position the country as one of the most biodiverse, accounting for 11,101 species of plants (Hassler, 1994). Costa Rica has eight indigenous cultures and a large number of protected areas, representing 25.26% of its terrestrial territory (Barrantes-Moreno, 2006). The first ethnopharmacology records of medicinal plants were made by European migrants, such as Monseigneur Bernardo Augusto Thiel in the 18th century, notorious for the report by indigenous people of cocoa’s butter capacity to act as snakebite antivenom when ingested during the fasting period (Hilje Quirós, 2020).

The systematic study of the flora of Costa Rica began in the first half of the 19th century, especially after the arrival of the Danish naturalist Anders Sandoe Oersted in 1846. However, scientific studies of medicinal plants started much later, beginning in the 1930s, particularly in the decades following the founding of the University of Costa Rica in 1940 (García-González and Morales, 2015). Recent surveys of the use of medicinal plants in Costa Rica showed that at least 107 species are currently used by the population (Hernández-Salón and León-Chavarría, 2023). Most of the recent publications available for medicinal plants are based on phytochemical and pharmacological studies. Phytochemical studies include the search for and identification of active or characteristic principles of plants; pharmacological works deal with the pharmacological actions and their possible mechanisms (García-González and Morales, 2015).

On the other hand, in Panama today, there is a strong influence of Spanish and indigenous cultures. There are seven recognized indigenous peoples of Panama: the Ngäbe, Buglé, Guna (Kuna), Emberá, Wounaan, Bri, and Naso Tjërdi. In 2010, they comprised approximately 12% of the total Panamanian population (IWGIA 2020), who are distributed in approximately 25% of Panama’s approximately 75,000 km^2^ that stretch between Costa Rica to the west and Colombia to the east.

Panama has an extremely diverse population of plants that have formed the basis of traditional medicine for the indigenous populations. A total of 10,444 species have been recorded (Correa and de Stapf, 2004) of which 916 have medicinal use, primarily for the digestive and respiratory systems (Torres et al., 2019). Both public (Ministry of Health and Social Security) and private healthcare services are provided in Panama, the latter representing private insurance carriers (Millett et al., 2024). Indigenous communities have little access to either system (Oestereich, 2020; PAHO, 2023), resulting in great reliance on traditional healing practices. In rural areas and cities, curanderos continue to provide local Panamanians with treatments prepared from local medicinal plants. The lack of health services in rural areas makes medicinal plants one of the most important resources (Torres et al., 2019), but in recent decades the market has been growing in both rural and urban areas.

Among the Tusipono Embera, herbal medicines are a primary choice in healthcare for uncomplicated illnesses, whereas conventional medicine is sought for more complicated issues (McWilliams et al., 2019). Among the Indigenous Naso of the jungle Bocas del Toro region along the Teribe river, every settlement has a *botánico* (herbalist) who knows the local flora. “What we cannot heal can be healed in the city, and what cannot be healed in hospitals, we treat with plants” describes the philosophy (Hawranek, 2020), underscoring the importance of both traditional and conventional healthcare for indigenous peoples.

The dietary supplement market in Pan America has produced increasing sales every year since 2008. In 2008, sales exceeded 1 billion USD, and in 2019, sales totaled more than 2 billion USD (Morán, 2020). In Panama, there is not a large commercial export market, but there are several notable exceptions including noni (*Morinda citrifolia* L.) and sarsaparilla (*Smilax aspera* L.) (Torres et al., 2019) for the dietary supplement market, ipecac (*Carapichea ipecacuanha* (Brot.) L. Anderson) for the conventional drug market, and Chinese banyan (*Ficus benjamina* L.) for the horticulture market.

### 2.3 South America

Argentina is a multicultural country with great ethnic diversity represented by 18 indigenous groups concentrated primarily in the northwestern Chaco and Patagonia regions. Traditional medicine is practiced among these groups by traditional healers and by common people who possess traditional knowledge and have access to medicinal plants. In many rural and peri-urban areas, people still utilize the services of local curanderos, *pay´e*, and medicos (healers), and there have been many interactions between traditional herbal medicine and formal biomedical services (Barboza et al., 2009). The Argentine flora includes more than 9,900 higher plants, of which approximately 1,600 are used medicinally, according to a survey conducted in 2009 (Zuloaga et al., 2008; Barboza et al., 2009; Rondina et al., 2010).

The herbal medicine tradition, as recorded in the earliest Argentinian manuscripts, reflects a mix of Indigenous Guarani, who occupied parts of Argentina, Paraguay, Uruguay, Brazil, and Bolivia, combined with Colonial European and current plant knowledge (Kujawska and Schmeda-Hirschmann, 2022). The first significant post-European contribution to medicinal plant studies in Argentina was in the early 18th century by Jesuits Pedro de Montenegro and Segismundo Asperger, who were described as “pioneers in the systematization of information relating to American pharmacopeia for their work editing catalogs of medicinal plants and their respective applications. The work itself was further described as containing “the unmistakable presence of Hippocratic and Galenic conceptions and a growing empiricism, characteristic of the scientific transformations seen in the 18th century” (de Montenegro, 1945; Fleck and Poletto, 2012).

Of particular interest was the recording of a relative lack of remedies available at the time the Jesuits presided, but this was undoubtedly an artifact of familiarity only with European remedies as well as a religious bias against the combined use of herbs and ritual that Jesuits considered demonic. Nevertheless, the recordings went on to list numerous plant-based remedies as being in use (Fleck and Poletto, 2012). Since then, many ethnobotanical studies have been conducted. One of the most prominent expeditions was the one undertaken by Alexander von Humboldt and Aimé Bonpland. The latter arrived in Argentina in the early 19th century and remained in the country for the rest of his life. He made invaluable contributions to the knowledge of the local flora (Mereles et al., 2020). In 1900, Dr. Juan A. Dominguez founded a museum (now called “Museum of Pharmacobotany”) based on his collections along with several well-known herbariums. His pioneering contributions to the Argentinian Materia Medica served as the foundation for the study of medicinal plants and the ongoing efforts to provide scientific support to their traditional use (Dominguez, 1928). A more recent comprehensive review of Argentinian medical botany is provided by Barboza et al. (2009) (Barboza et al., 2009); in 2013, an estimated 1,529 species of medicinal plants were recorded as being used, most of which have not been subjected to in-depth chemical and pharmacological investigation (Vattuone, 2013).

In contrast to other regions of Latin America, with some exceptions, the indigenous medicinal plant knowledge base of Argentina was not very well developed prior to the arrival of Europeans or was considered so as an artifact of European prejudices. Subsequently, European occupation greatly influenced medical practices in favor of those developed in Europe. The complete extinction of whole groups of indigenous peoples, such as the Tonocotés, Lule-Vilela, Sanavirones, and Chana-Timbúes in Argentina, undoubtedly resulted in a great loss of medicinal plant knowledge (Montenegro and Stephens, 2006). In some primary regions of commerce and colonization (e.g., the Pampa regions), a great deal of assimilation has occurred among the indigenous people. While it remains common to seek the help of a curandero, the treatments provided may be more common current practice of herbal medicine (Villamil, 2004). In rural communities, a spectrum of healthcare options is available, ranging from consultations and treatments by curanderos to conventional medical care (Trillo et al., 2010).

A survey of six villages of the Arid Chaco forest of western Cordoba Province identified the medicinal uses of 117 native and 34 exotic plant species. The uses included digestion (49%), external use (14%), respiration (14%), diuresis (8%), circulation (9%), sleep (2%), ritual (1%), and gynecology (2%), among others (1%). One seminal finding of this survey was that at least part of the medicinal knowledge appears rooted in the Spanish (criollos) culture, providing a continuity of medicinal plant use that was influenced greatly by early Spanish settlers. Like other Meso-American cultures, an emphasis was placed on healing or otherwise addressing physical, emotional, mental, spiritual, and environmental health. Another interesting finding from the same survey was that women had two to three or more times greater usage of traditional healing practices than men (Trillo et al., 2010). The use of medicinal plants by midwives in traditional communities is also relatively common (Martínez, 2008).

A survey of retail herb stores and pharmacies found that cultural orientation, more than economics, influenced the use of herbal medicines. The most popularly used botanicals were malva or mallow (*Malva* spp.) (18%), manzanilla or chamomile (*Matricaria chamomilla* L) (13%), tilo or linden (*Tilia* spp.) (12%), cuasia (*Picrasma crenata* (Vell.) Engl.)) (8%), and boldo (*Peumus boldus* Molina) (7%) (Bach et al., 2014). In some communities there is a specific use of medicated honeys, approximately 50% of which are mixed with botanicals (Kujawska et al., 2012). The current motivators for herbal use are traditional precedent, popular commercial products, ancestral use, cost-effectiveness relative to conventional pharmaceuticals, and a perception that botanicals are a safer alternative to conventional drugs. As in other countries, herbal medicines reflect a mix of cultures, with *The National Pharmacopoeia* including 31 plant drug monographs but only four monographs for native medicinal species (Gobierno de Argentina, Administración Nacional de Medicamentos, Alimentos y Tecnología Médica, 2024). Additionally, there are some provincial programs, such as *Cultivating the Health* under the auspices of the Argentinian Phytomedicine Association, that are designed to provide free healthcare to the underserved, specifically by promoting the use of medicinal plants in primary healthcare. Some botanicals have been provided to communities via primary healthcare centers (Alonso, 2005).

In addition to the current mix of herbal medicine traditions based on historical precedent, Ayurvedic herbal traditions of India have spread throughout Latin America, including Argentina. In the 1990s, the Fundación Salud de Ayurveda Prema Argentina promoted Ayurveda. Based in Buenos Aires, the foundation, in collaboration with Gujarat Ayurveda University, India, offered courses in Ayurveda in two of the country’s major medical schools, the School of Medicine of the University of Buenos Aires and the National University of Cordoba’s School of Medicine (Alonso, 2005).

In the case of Chile, prior to the arrival of the Spanish, there were several indigenous groups inhabiting this South American country, the largest of which was the Mapuche (people of the earth), who primarily lived in south-central Chile and extended into south-western Argentina. Other groups of people that are identified in this region correspond to the Aymara, Rapanui, Atacameño or Likan Antai, Quechua, Colla, Chango, Diaguita, Kawésqar, and Yagán (CONADI, 1993). Recent archaeological evidence from South America suggests that the migration from Asia to America might have taken place much earlier. This evidence comes from the Brazilian site of Boqueirao do Sitio da Pedra Fur ad a, with a long cultural timeline possibly extending as far back as 32,000 years BP, and the Chilean site of Monte Verde. Archeological records suggest the area was occupied since at least 600 to 500 BCE (Dillehay and Collins, 1988; Trovall, 2017).

The Mapuche have a very sophisticated hierarchy of traditional healers that range from herbalists, to surgeons, to priest-shamans, whose knowledge was obtained through study and constant observation. In medicinal plants reports from Chile, the efforts of Adolfo Murillo, who addressed the medicinal properties of Chilean plants from their botanical categorization, are particularly noteworthy. Also significant is the work of Amador Guajardo, who made a summary of the healing vegetables of Chile, locating their biogeographical distribution, explaining their uses, and reviewing their history. An example is natre (*Solanum crispum* Ruiz and Pav), a natural and effective ancient agent used as an antipyretic.

In the earliest days of colonization, the Mapuche used approximately 200 plants as medicines (Otero et al., 2023). A current online version of the Chilean Flora similarly lists 200 medicinal plants (Belov, 2023). The first Chilean Pharmacopoeia was established in 1882, and like most pharmacopoeias of the period, was dominated by plant-based preparations, including several native plants such as *P. boldus* Molina, *Luma chequen* (Molina) A. Gray, *Buddleja globosa* Hope, *Drimys winteri* J.R. Forst. and G. Forst., *Quillaja saponaria* Molina, and *Aristotelia chilensis* (Molina) Stuntz. In the fourth and current edition of the Chilean pharmacopoeia, the aforementioned species persist, and others have been added, such as *Haplopappus baylahuen* J. Rémy, *Fabiana imbricata* Ruiz and Pav, and *Ugni molinae* Turcz. In addition, it contains some species of traditional use in the country that were introduced from Europe.

In Chile, there are several initiatives designed to cultivate an integration of Mapuche healing practices into some primary care settings [e.g., Programa de Salud Mapuch (Mapuche Health Program) and the Mesa Local (PROMAP)]. For example, a resurgence of natural remedies occurred around 2003 when a Mapuche community took over the administration of the Maquehue Hospital and Health Centers of Boroa Filulawen and Ñi Lawentuwün in Araucanía Region. In addition to the above, the Special Program for Health and Indigenous Peoples (PESPI) and the Origins Program focused on an intercultural health model (Poblete, 2019).

The Ministry of Health of Chile provides some technical guidance documents on the cultivation, harvesting, drying, packaging, dispensing, and use of traditional herbal medicines, which correspond to native and introduced herbs that can be used with therapeutic properties in the form of infusions (Republica de Chile, 2018). In 1998 in the Ngawbere territory, a variety of traditional healers were formally trained as herbalists or health assistants; they resided in the village and provided varying degrees of care to indigenous communities (Bletzer, 1998). In relatively recent years, a new ethnobotanical database, Rizoma Database of native Chilean plant medicines, was launched and presents information from 1,380 records about the use of 736 vascular species (Cordero et al., 2022).

Only a few native Chilean herbs appear prominently in international commerce, namely, boldo (*Peumus boldus*), maqui berry (*Aristotelia chilensis*), and soapbark tree (*Quillaja saponaria*). Boldo was included in The Dispensatory of the United States of America from 1878 to 1947; maqui berries emerged in the United States as a “superfood” in recent decades, and soapbark tree was listed in early editions of USP from 1880 to 1900 (Boyle, 1991).

Brazil possesses a widely diverse flora comprised of approximately 45,000 species that make up 20%–22% of the world’s higher plants, including a plethora of which are used medicinally (Dutra et al., 2016). In Brazil, the first description of the use of plants as medicine was made by Gabriel Soares de Souza, author of the Descriptive Treated of Brazil in 1587. This treated described the medicinal products used by the Indians as “the trees and herbs of virtue”. When the first Portuguese doctors arrived in Brazil, faced with the shortage of medicines used in Europe in the colony, they realized the importance of plants used by indigenous people as medicine (De Sousa et al., 1971; Veiga et al., 2002; Argenta et al., 2011).

Notable Brazilian plants that have been included in both traditional and modern materia medica include *C. ipecacuanha* (ipecac) that has been a common emetic used in modern medicine until relatively recently, *Pilocarpus microphyllus* Stepf. ex Wardlex, the muscarinic acetylcholine agonist used as an adjunct in glaucoma treatments (Nogueira et al., 2010), and more recently, açaí (*Euterpe oleracea* Mart.), a native fruit from the Amazon rainforest that has emerged as a common dietary supplement ingredient worldwide (Carey et al., 2017; Lepsch-cunha et al., 2023).

Despite the great diversity, the native vegetation of Brazil has been subject to widespread destruction succumbing to a variety of national agricultural and economic pressures that have resulted in the replacement of megadiversity with monocultures of food commodities. This has been somewhat offset by the development of bioproducts from Brazilian plants that continue to pit native biodiversity against even sustainable agricultural initiatives (Palhares et al., 2020). Providing formal recognition for the potential benefits of medicinal plants has been proposed as a strategy for the preservation of natural resources (Fernandes and Boff, 2017).

Community markets continue to serve a role in providing information regarding the medicinal use of plants to consumers (Monteiro et al., 2010). According to one survey, market vendors reported that 91 species were used medicinally as decoctions, infusions, and sauces for 291 indications (de Carvalho et al., 2015), while another survey reported flu, inflammation, and stomach problems as among the most common indications for which plants are used (Magno-Silva et al., 2021). Medicinal plants used can be categorized as; i) native species that are collected in the local ecosystems and available through local markets, ii) cultivated exotic species, and iii) imported species (mainly dry plants and extracts), these latter two categories consisting predominantly of commercial products available in pharmacies and natural products stores (Palhares et al., 2020). At the same time, there has been a tremendous amount of research on the bioprospecting of medicinal plant compounds for the potential development of modern drugs. Between 2011 and 2013, more than 10,000 scientific papers on various aspects of Brazilian plant research were published (Dutra et al., 2016).

Colombia has one of the richest and floristically diverse ecosystems on earth, with an extensive history of traditional medicine in which medicinal plants are integral. The country is home to approximately 10% of the world’s biodiversity, consisting of an estimated 26,000 to 50,000 plants, of which 115 were included in the *Colombian Vademecum of Medicinal Plants*, a document initiated by the Colombian Ministry of Social Protection in 2008 (Gómez-Estrada et al., 2011; Bernal et al., 2019). Indigenous communities, such as the Kogui, Wiwa, and Arhuaco, have been using medicinal plants for centuries to treat various ailments and diseases. Many of Colombia’s traditional medicines continue to be used in ways that are consistent with historical practices.

Along with its floral diversity, Colombia is characterized by great topographic, climactic, and cultural diversity encompassing the mountainous Andes, lush Amazon rain forest, tropical Caribbean coastlines, and lowlands, along with all the plants and people that inhabit these diverse regions. According to the work of Dr. Pérez Arbeláez (1896–1972) from the National University of Colombia, it is necessary to make a clear distinction between the herbal medicine traditions of the Amazonian rain forest and those used throughout the rest of Colombia (Perez-Arbeláez, 1956).

Investigators researching rainforest dwellers have placed significant emphasis on traditional drugs such as curare, the paralyzing drug derived from *Chondrodendron tomentosum* Ruiz and Pav, and hallucinogens (e.g., ayahuasca). Although a great deal of other work collecting medicinal plant data has been undertaken, it has not been subjected to in-depth experimentation, leaving much of Colombia’s medicinal flora uninvestigated. The basis of medicinal plant use throughout the rest of Colombia is best represented by the ancient *Chibcha* (*Muisca*), who, before Spanish conquest, occupied the regions around the current cities of Bogota and Tunja.

Throughout Colombia’s history, including periods of colonization, and despite a struggle for some communities to maintain their cultural identity, a rich and diverse cultural use of medicinal plants has been maintained. Many species of plants are still used widely to treat common illnesses throughout most of Colombia (Gómez-Estrada et al., 2011; Bussmann et al., 2018) and include both native and introduced species (Cadena-González et al., 2013). Some communities predominantly use introduced plants. The List of Medicinal Plants Approved for Therapeutic Purposes of the National Institute of Food and Drug Surveillance (INVIMA: Instituto Nacional de Vigilancia de Medicamentos y Alimentos (Colombia), 2024) contains 196 species. The Ministry of Health defers to the Caribbean Herbal Pharmacopoeia of TRAMIL, which provides a basic assessment of the safety and effectiveness of traditional herbal medicines (TRAMIL, 2017).

Peru is a diverse country with 84 of the 103 biospheres on the planet and 28 of the 30 existing climates in the world, placing it among the 20 countries with the greatest biodiversity on Earth (Pereyra de Pribyl, 2013). Its 1.2 million square kilometers consist of coast, mountainous, and jungle regions. Peru’s current population exceeds 31.5 million, which includes 4 million indigenous peoples. In addition, 80% of the total population self-identify as either indigenous or mixed (mestizo) (International Working Group for Indigenous Affairs, 2023).

The use of medicinal plants is an important component of traditional Peruvian medicine and has persisted since pre-Incan times, with some plants recorded in ancient ceramics, textiles, and cave drawings, as well as through the writings of early Dominicans and Jesuits. In addition to the New World food plants (corn, squashes, beans), Peru gave to the world cinchona bark, from which the antimalarial drug quinine was derived. Today, more than 70% of people seeking medical care have used a medicinal plant. In indigenous communities, medicinal plants are often the only available therapeutic option.

Conversely, it is also reported that “the health-seeking behavior of the Andean households is independent of the degree of availability of biomedical facilities in terms of quality of services provided, physical accessibility, and financial affordability,” and “preference for natural remedies over pharmaceuticals coexists with biomedical healthcare that is both accessible and affordable.” Additionally, greater access to biomedicine did not lead to less prevalence of Andean indigenous medical knowledge (Mathez-Stiefel et al., 2012).

Some key medicinal plants are exported from Peru internationally, most notably maca (*Lepidium meyenii* Walp.), cat’s claw (*Uncaria tomentosa* (Willd. DC), and stone breaker (*Phyllanthus niruri*) for dietary supplements. Also native to Peru are the illicit drug cocaine, derived from the leaves of coca (*Erythroxylum coca* Lam.), but otherwise beneficial herb for the treatment of altitude sickness and a traditionally used local anesthetic, and dragon’s blood (*Croton lechleri* Müll. Arg.), which is the source a recently U.S.-approved botanical drug (Bussmann, 2013). The hallucinogenic Ayahuasca, also from Peru, has made a significant impact on nations worldwide, primarily for its ceremonial and recreational uses (Jiménez-Garrido et al., 2020).

Ecotourism that includes the study of herbal medicine among traditional healers occurs in Peru, as in other regions (Aracari Travel, 2014). There are a limited number of available plant medicines in this program, and reportedly, are focused on those subjected to “rigorous testing” for safety and efficacy. They are supplied in part through a network of botanical farms that were established as part of this initiative decades ago.

Health officials in Peru partnered with the WHO/PAHO to provide the scientific basis for offering specific medicinal plant preparations through their clinics (Vallejos-Gamboa et al., 2020). Subsequent outcomes research conducted annually revealed a high level of consumer satisfaction with the natural therapies, as well as objective improvements in terms of disease reduction and decreased use of pharmaceutical medications at a lower cost. However, patients were “happy to use both medicinal plants and pharmaceuticals” (Palmer, 2019).

## 3 Regulation and integration of herbal products into health systems

There are various regulatory models governing the trade of herbal products, as well as different levels of integration of herbal products into national healthcare systems internationally. In most countries, herbal products used for therapeutic purposes are regulated as herbal medicines, herbal drugs, or traditional medicines. In a few countries, herbs are either exclusively or additionally traded as “supplements.” Several categories of products are considered in regulation, namely: 1) commercial products, 2) traditional herbal remedies used by indigenous healers, and 3) herbal products dispensed or prescribed by health practitioners.

Herbal products and natural health practitioners are regulated and integrated differently throughout the Pan American region, with some countries integrating both with varying level of support from regulators. The development of herbal ingredient monographs typical of formal pharmacopoeias is specific to the modern herbal products with ingredients and dosage forms used in modern healthcare (tablets, pills, capsules, etc.) but may not be applicable to the most traditional of herbal products and ingredients traded in markets or dispensed by traditional healthcare practitioners. Yet, all categories of herbal products can benefit from the quality standards of identity, strength, purity, and limits on contaminants that are provided by pharmacopoeial monographs. The primary factors for determining the level of integration into national healthcare systems include cultural orientation for or against herbal medicines and access to modern healthcare services. Supplementary Table S2 contains a representative sample of Botanical Dietary Supplements and Herbal Medicine regulations, terminologies and definitions, and evidence of integration into National Health Systems in countries of the Pan American regions. This is not meant to be an exhaustive review of the regulatory status of herbal products in every country throughout Pan America. Rather, it is meant to represent the variety of legal frameworks that exist within the region. The information presented is based on a review of publicly available documents, including WHO/PAHO/BIREME Virtual Library and Americas Network in TCIM resources (WHO/PAHO/BIREME Virtual Health Library TCIM Americas and TCIM Americas Network, 2024), and the input from the USP BDSHM Pan American Expert Panel members.

In the United States, most herbal products are classified as dietary supplements, which for statutory purposes are a category of food. Based on the Dietary Supplement Health and Education Act of 1994 (DSHEA), the Food and Drug Administration (FDA) regulates dietary supplements under a different set of regulations from those covering “conventional” foods and drug products (U.S. National Institutes of Health, Office of Dietary Supplements, 2024). Under current U.S. law, herbal supplement manufacturers have a legal and regulatory responsibility to maintain product quality and safety, identify and characterize new dietary ingredients with a pre-market notification to FDA, label their products in conformity with legal requirements, adhere to Current Good Manufacturing Practices (GMPs) (21 CFR 111), and report serious adverse events to FDA’s MedWatch program (U.S. National Archives, 2024; U.S. Congress, 2002; U.S. Congress, 2006). These aspects are overseen by numerous regulatory bodies including state and federal health boards, the FDA, the Department of Homeland Security, and the Federal Trade Commission (Avigan et al., 2016).

Traditional Chinese medicine (TCM), predominantly acupuncture, is allowed in 49 U.S. states (Suh, 2020), while naturopathic medicine is allowed in 26 jurisdictions (23 states and three territories) (Cody, 2019). Naturopathic medicine is a distinct primary healthcare system that blends modern scientific knowledge with traditional and natural forms of medicine. In all states that license naturopathic medicine, naturopathic physicians are authorized to dispense herbal products to their patients after a consultation has been made. Of states that license the practice of TCM, only four specifically provide authorization for the use of herbs, and only as “dietary supplements.” Herbal products may not be dispensed for the treatment or prevention of disease, even though these practitioners are regarded as primary healthcare providers. U.S. practitioners who manufacture their own products must technically meet all GMPs and federal regulations for dietary supplements.

In Canada, herbal health products are a category of non-prescription drugs, governed by the Natural Health Products Regulations enacted in 2003 (Government of Canada, 2003). Natural Health Products (NHPs) encompass various materials including plant, algal, bacterial, fungal, and non-human animal materials, along with extracts, isolates, vitamins, minerals, amino acids, fatty acids, and probiotics. Homeopathic and traditional medicines are explicitly included, and multiple routes of administration (e.g., topical or oral) are allowed, except for injection. The legal sale of NHPs in Canada requires pre-market approval in the form of a product license based on evidence for efficacy supporting self-care health claims on the label, ensuring safety and quality. Canadian sites that manufacture, package, label, and/or import these products must have a site license issued by Health Canada based on the evaluation of submitted evidence of compliance with Good Manufacturing Practices. Although there is no requirement that firms engaged solely in the distribution of NHPs possess a site license, these firms are expected to comply with the elements of Good Manufacturing Practices (GMP) relevant to distribution activities.

In both Canada and the United States, there are varying degrees but relatively little integration of herbal medicine into modern medical practice, and almost none within the insurance reimbursement system. In the United States, there is a regulatory model for obtaining approval of prescription botanical drugs through a process initiated by the FDA (U.S. Food and Drug Administration, 2016). However, to date, only two botanical drugs have been approved, both of which are for indications for which no other therapeutic options exist. The two drugs are Veregen^®^ sinecatechins ointment (approved in 2006), a purified extract of green tea used for the topical treatment of genital warts, and crofelemer (Mytesi^®^), an extract of latex of dragon’s blood (*C. lechleri*), approved in 2012 for intractable diarrhea in patients with acquired immunodeficiency syndrome (AIDS) for which no other treatment has been successful. Additionally, there are some OTC drugs that are derived from botanical ingredients that have persisted in the market as “grandparented” drug ingredients (e.g., *Senna alexandrina* Mill) (Brinckmann et al., 2020). In Canada, there is also a regulatory process for market authorization of botanical drugs, but there are only a few legacy prescription botanical products on the Prescription Drug List (Health Canada, 2023): foxglove (*Digitalis lanata* Ehrh. and *D. purpurea* L.), Indian snakeroot (*Rauvolfia* spp.), and white and green hellebore (*Veratrum album* L. and *V. viride* Aiton).

In Canada, of the 10 provinces and three territories, five provinces and 1 territory regulate the practice of naturopathic doctors through the following laws: British Columbia (*Health Professions Act Naturopathic*, 2008), Alberta (*Naturopaths Profession Regulation, Alta*, 2012), Saskatchewan (*The Naturopathy Act*, 1974), Manitoba (*The Naturopathy Act*, 1988), Ontario (*Health Professions Act*, 1991), and the Northwest Territories (*Health and Social Services Professions Act*, 2022). *The Naturopathic Doctors Act* of 2008 grants title protection for naturopathic doctors in the province of Nova Scotia. Naturopathic doctors are actively engaged in the process of seeking regulation in the other provinces and territories. Two provinces, British Columbia and Ontario, regulate both TCM practitioners and acupuncturists (*Traditional Chinese Medicine Act*, 2006, and *Health Professions Act: Traditional Chinese Medicine Practitioners and Acupuncturists Regulation*). Alberta, Quebec, and Newfoundland and Labrador regulate acupuncturists only (France, 2022). Finally, homeopaths are regulated in Ontario only (de Rege, 2023).

Since 2018, COFEPRIS (Mexico’s Federal Commission for Protection from Sanitary Risks) has been the organization responsible for regulating, controlling, and promoting health and safety in Mexico (Estados Unidos Mexicanos - Cámara de Diputados, 2024a; Estados Unidos Mexicanos - Secretaría de Gobernación, 2021; Estados Unidos Mexicanos - Consejo de Salubridad General, 2018a; Estados Unidos Mexicanos - Consejo de Salubridad General, 2021a). It oversees the registration, importation, and exportation of various products, including drugs, foods, beverages, cosmetics, biotechnological items, and medical devices. Botanical products are categorized as herbal medicines and herbal remedies and are presented in a pharmaceutical form, with quality control parameters specified in the *Mexican Herbal Pharmacopeia* (Comisión Permanente de la Farmacopea de los Estados Unidos Mexicanos, 2024); they may not contain psychotropic or allopathic drugs (Estados Unidos Mexicanos - Presidencia de la República, 2024b). Another category includes homeopathic drug products with herbal ingredients per the *Mexican Homeopathic Pharmacopoeia*. Lastly, there are food supplements, comprising vegetal products, traditional foods, or fruit extracts with added vitamins or minerals, presented in a pharmaceutical form, for dietary enhancement or substitution (Estados Unidos Mexicanos - Presidencia de la República, 2024a).

Even though the use of these products is limited in the healthcare system, consumers and practitioners use the above products and plants that are available in rural areas according to their traditions, or these are sold in the markets, which are scarcely regulated. After 2018, the evaluation is performed by a committee for herbal medicine, and it may require the presentation of clinical studies if necessary (Estados Unidos Mexicanos - Consejo de Salubridad General, 2018; Estados Unidos Mexicanos - Consejo de Salubridad General, 2023). On the other hand, the growth of the Mexican herbal market is in part fueled by the longstanding traditional integration of medicinal plants in domestic and traditional healing practices and part due to the lower expense of herbal preparations compared to modern medications and treatments.

Central American countries, comprising Belize, Guatemala, Costa Rica, El Salvador, Honduras, Nicaragua, and Panama, constituted the Central American Integration System (SICA), which was also joined by the Dominican Republic. As part of their integration strategy, the Central American Technical Regulations (RTCA) aim to establish, among others, the conditions and requirements for the sanitary registration of medicines for human use, including products of botanical origin. These RTCA address specific aspects related to health registry requirements, quality verification, and labeling requirements for pharmaceutical and medicinal products in the region. In the case of herbal medicines as natural medicinal products, three technical regulations are established: RTCA 11.03.64: 19 Requirements for Health Registry; RTCA 03.11.56: 09 Quality Verification; and RTCA 11.04.41: 06 Labeling Requirements (Jeimy et al., 2016; COMIECO, 2019).

For instance, in Guatemala, medicinal plants were regulated by Decree No. 90-97, and since September 2021 they are regulated at the regional level under the above mentioned RTCA. Some traditional healers in Guatemala have specific medicinal specialties, and the use of herbs and natural products is common throughout the country (Gobierno de la Republica de Guatemala, 2018). In 2006, a list of 101 plants was compiled and incorporated into a *National Vademecum of Medicinal Plants*, particularly to serve the healthcare needs of those in rural communities (Cáceres, 2009). Additionally, the Ministry of Public Health has a Commission for Traditional and Alternative Medicines that works to integrate some alternative health activities into the public health system. In 2022, the Vademecum was officially accepted as a list, and Instructions for Use were provided for 49 medicinal plants to be used in support of primary health attention for COVID-19 patients (Cáceres and Cáceres, 2019; REGMIC, 2020).

In Costa Rica, RCTA were established by Executive Decree No. 37851 in 2013 as Resolution N° 303-2013 (COMIECO-EX). Medicinal plants are not included officially in the public health system, but some initiatives within the Costa Rican Social Security Fund (CCSS), the institution that oversees the public health hospitals and clinics, allocated some offices for the eventual consultation of indigenous therapists and herbal care at the Suretka-Talamanca Clinic beginning in 2000 (López et al., 2019).

Medicinal plants are regulated in Panama by regulatory agencies such as the National Directorate of Pharmacy and Drugs, the Ministry of Health, and the Department of Food (DEPA) at the Ministry of Agriculture. These agencies have passed several laws to regulate botanical dietary supplements and herbal medicines (Ministry of Health of the Republic of Panama, 1990). Executive Decrees 852 and 854 established RCTA as the guidelines for labeling and verification of quality of natural products respectively. The use of natural products and botanical dietary supplements has made its way into the national health primary care system (Caballero-George and Gupta, 2011) and is as prevalent as modern medicine (Peddicord, 2023). However, due to generational practices and lack of coverage, many indigenous populations, such as Ngöbe-Buglé, Kuna, Emberá-Wounan, Naso or Teribe, and Bri-Bri, rely on traditional medicine. The rise of naturopathic medicine is also reaching Panama, with centers created to focus on holistic healing (Gupta et al., 2005; Cortez, 2013).

In the Caribbean, the TRAMIL network, as mentioned, has validated and expanded health practices based on the use of medicinal plants (Alvarado-Guzmán et al., 2009). In fact, the TRAMIL monographs are used by the ministries or departments of health in countries in the region as a reference and for the training of health professionals. According to TRAMIL methodology, monographs include scientific data and recommendations for use, the plant species, plant part, and form of preparation of the traditional remedy to update and train primary healthcare professionals about the medicinal plant’s significance. Each significant use is validated for safety according to standards and protocols of the Centers for Disease Control (CDC) and the Organization for Economic Co-operation and Development (OECD), as well as efficacy following *in vivo* and *in vitro* protocols (Alvarado-Guzmán et al., 2009). The TRAMIL monographs, however, lack the quality control guidance regarding identity, strength, purity, and limit of contaminants that is typical of pharmacopoeial monographs.

In Argentina, medicinal plants fall within four categories, and their regulation and control are managed by health authorities of the Drug, Food and Medical Technology National Administration (ANMAT). Herbal medicines (Gobierno de Argentina, Ministerio de Salud y Accion Social, 1998; Gobierno de Argentina, Administración Nacional de Medicamentos, Alimentos y Tecnología Médica, 1999a; Gobierno de Argentina, Administración Nacional de Medicamentos, Alimentos y Tecnología Médica, 1999b; Gobierno de Argentina, Administración Nacional de Medicamentos, Alimentos y Tecnología Médica, 2000; Gobierno de Argentina, Administración Nacional de Medicamentos, Alimentos y Tecnología Médica, 2004; Gobierno de Argentina, Administración Nacional de Medicamentos, Alimentos y Tecnología Médica, 2008; Gobierno de Argentina, Administración Nacional de Medicamentos, Alimentos y Tecnología Médica, 2015a; Gobierno de Argentina, Administración Nacional de Medicamentos, Alimentos y Tecnología Médica, 2015b; Gobierno de Argentina, 2020), cannabis products (Gobierno de Argentina, Administración Nacional de Medicamentos, Alimentos y Tecnología Médica, 2022), and cosmetics (Gobierno de Argentina, Administración Nacional de Medicamentos, Alimentos y Tecnología Médica, 1999c) constitute three categories. The last category is dietary supplements considered as foods, which are elaborated with a pharmaceutical form and intended for healthy patients who might need to improve a specific nutritional intake.

The first two categories share the same quality standards as the rest of the pharmaceutical products sold in Argentina, and the facilities that manufacture them are subjected to frequent quality inspections by health authorities. Within the formal healthcare system, there are many pharmaceutical products based on medicinal plant extracts that physicians prescribe instead of synthetic active pharmaceutical ingredients The Argentinian National Pharmacopoeia includes 31 plant drug monographs and 4 for medicinal native plants. Since no herbalists or herbal doctors exist as such, herbal practitioners lack formal training and work outside the formal health system (Gobierno de Argentina, Administración Nacional de Medicamentos, Alimentos y Tecnología Médica, 2024). However, some physicians use herbal products in their practices to complement and/or replace conventional therapeutic strategies based on pharmaceutical products. Some examples are Senna leaves, St. John’s wort, Asian ginseng, soy isoflavones, chia seeds, blueberry dry extract, psyllium seeds, and ginkgo; these may be prescribed by physicians, although the use of these types of products is limited.

In Argentina, there are several initiatives aimed at improving healthcare access, specifically through the integration of natural therapies into primary healthcare. The Provincial Ministry of Health has provided training to health personnel, farmers, and the general population about the rational and responsible use of medicinal plants (Moreau, 2006; Kujawska et al., 2012). There is also a Traditional and Alternative Medicine Program that, in addition to educating people about herbs used in local communities, also provides education about the benefits of Ayurvedic and Chinese medicine in public health. In some areas (e.g., Rosario City), there are city ordinances to “guarantee access to traditional medicines, promote their rational use … and promote a greater contribution by traditional medicine to the public health system” (Thornett, 2016).

In Chile, the products related to medicinal plants must have a health registry and be governed by regulations of the National Control System of Pharmaceutical Products for Human Use. The main purpose of the Department of Health and Indigenous Peoples and Interculturality of the Ministry of Health of Chile (MINSAL) is to formulate and adapt health policies, standards, and programs with cultural relevance aimed at overcoming health inequities of indigenous peoples and other culturally diverse populations that coexist in the country, within the framework of respect for their rights and guaranteeing their participation (Republica de Chile. Ministerio de Salud Division Juridica, 1996; Republica de Chile de Salud et al., 2010). On the other hand, the Department of Pharmaceutical Policies and Regulations, Health Providers and Complementary Medicines of the MINSAL fulfills normative and regulatory roles and, in the field of complementary medicine, addresses matters related to the knowledge, recognition, and practice of complementary/alternative medicines.

Medicinal plants in Colombia are regulated by INVIMA (INVIMA. Colombia, 2023, 2023). Sanitary registration is mandatory for pharmaceutical preparations based on medicinal plants and traditional phytotherapeutic products to ensure compliance with regulatory requirements and good manufacturing practices (Gobierno de Colombia, 2004; Gobierno de Colombia, 2005; Gobierno de Colombia, 2018). The Colombian government approves the use of medicinal plants as alternative medicine. In 1994, the Committee for Review of Pharmaceutical Products produced a list of approved medicinal plants and their uses. The *Colombian Vademecum of Medicinal Plants*, created by the Social Protection Department, provides information on approved species, pharmacological activity, constituents, usage instructions, toxicity, contraindications, and available dosage forms (Gobierno de Colombia, 2008; Gómez-Estrada et al., 2011). Colombia’s Vademecum is comprised predominantly of introduced plants (Duque et al., 2018).

To develop drugs from a genetic resource in the region, manufacturers are required to enter into a contract with the Colombian Ministry of the Environment for scientific research on DNA/RNA isolation, secondary metabolites isolation, and patent request based on the Convention of Biological Diversity of 1993 and the Andean Decision 391 from Cartagena signed in 1996 by Bolivia, Colombia, Ecuador, Peru, and Venezuela (Trujillo et al., 2009). Despite these initiatives, there is very little integration of traditional herbal medicines into the national healthcare system, underscoring that most traditional use is relegated to either self-care or care provided by traditional healers.

Considering Brazil, in 1994 the Brazilian Ministry of Health established its first directive to evaluate the safety, quality, and efficacy of marketed herbal drugs that predominantly followed WHO and select European guidelines. As such, herbal drugs are registered as medicines and must be determined to be safe and effective for their intended use and produced according to national quality control standards. In 2016, the Brazilian market for herbal medicines was estimated at $261 million (US) (Dutra et al., 2016) that includes consumption of Brazil nuts for reducing markers of inflammation (Colpo et al., 2014), the antihypertensive effects of chia seeds, and the use of various herbaceous species of *Phyllanthus* for antispasmodic and analgesic activity used in the treatment of urinary and gallbladder complaints. Despite promising clinical and pre-clinical findings, very few native Brazilian plants have been adequately assessed clinically (Dutra et al., 2016). Relatively recently, regulatory changes were implemented that recognize herbal medicines and traditional herbal products as the primary regulatory categories for herbal products. As of 2018, there were 332 single and 27 combination registered herbal medicine products (Carvalho et al., 2018). As in other countries, despite regulatory controls, problems regarding adulterations, substitutions, and contaminations of herbal products continue to exist underscoring the continued need for guidance such as is provided by pharmacopoeial monographs (Palhares et al., 2020).

Brazil has had a National Policy and its own Medicinal Plants and Herbal Medicines Program since 2006 and 2008. The Policy’s general objective is to guarantee the Brazilian population safe access and rational use of medicinal plants and herbal medicines, promoting the sustainable use of biodiversity, and the development of the production chain and industry. Its goal is to expand therapeutic options for users, ensuring safety, efficacy and quality, consideration of traditional knowledge about plants, construction of the regulatory framework for production, distribution and use of medicinal plants and herbal medicines based on existing models and experiences in Brazil and other countries. Among its objectives are: the promotion of research and development focused on technologies and innovations in medicinal plants and herbal medicines in the different phases of the production chain; promoting the sustainable development of medicinal plants and herbal medicine production chains and strengthening the national pharmaceutical industry in this field; promoting the sustainable use of biodiversity and sharing the benefits arising from access to genetic resources of medicinal plants and associated traditional knowledge. The Program and the National Committee for Medicinal Plants and Phytotherapeutics aim to regulate the production, use and distribution of medicinal plants (Brazil. Ministério da Saúde, 2006; Brazil. Ministério da Saúde, 2008; Brazil. Ministério da Saúde. Agência Nacional de Vigilância Sanitária–ANVISA, 2014c; Brazil. Ministério da Saúde. Agência Nacional de Vigilância Sanitária–ANVISA, 2014a; Brazil. Ministério da Saúde. Agência Nacional de Vigilância Sanitária–ANVISA, 2014b; Brazil. Ministério da Saúde. Agência Nacional de Vigilância Sanitária–ANVISA, 2015; Brazil. Ministério da Saúde. Agência Nacional de Vigilância Sanitária–ANVISA, 2019; Brazil. Ministério da Saúde. Agência Nacional de Vigilância Sanitária–ANVISA, 2020; Brazil. Ministério da Saúde. Agência Nacional de Vigilância Sanitária–ANVISA, 2022).

From an intersectoral perspective within the Ministry of Health, the Secretariat of Primary Healthcare, in 2006, included phytotherapy/medicinal plants in the National Policy of Integrative and Complementary Practices in the Unified Health System, as one of the modalities of health practices, in the sense to promote greater incorporation and access to the use of phytotherapy in Brazil (Brazil. Ministério da Saúde. Secretaria de Atenção à Saúde, 2006).

Medicinal plants are integrated into the Brazilian national health system, through the National List of Essential Medicines (RENAME), the National List of Plants of Interest for the SUS (RENISUS), the Brazilian Pharmacopoeia (FB) and the Phytotherapeutic Formulary (FFFB) (Brazil. Ministério da Saúde. Secretaria de Atenção à Saúde, 2006). In these species lists and botanical monographs published by the National Health Surveillance Agency (ANVISA) and the Ministry of Health (MS). Through ANVISA regulations, the following enter the national production system: *Mikania glomerata* Spreng. (guaco), *Maytenus ilicifolia* Mart. ex Reissek (espinheira-santa or holy thorn), representatives of the flora of southern Brazil, *Myracrodruon unrudeuva* M. Allemão (aroeira-do-sertao) and *Lippia sidoides* Cham. (pepper rosemary), both representing the Northeast, *Schinus terebinthifolia* Raddi (beach pepper tree), which represents the Atlantic Forest, *U. tomentosa* (Willd. ex Schult.) DC (cat’s claw) and *Piper hispidinervum* C. DC (valuable safrole-producing long pepper from the Amazon), *Mentha* x *villosa* Huds. (creeping mint) and *P. niruri* L. (pombinha or stonebreaker herb), both occurring throughout the national territory, are some expressive examples.

Medicinal plants in Peru are regulated by the provisions of the Law on Pharmaceutical Products, Medical Devices and Sanitary Products and its Regulations, approved in 2009, and a Supreme Decree approved in 2011 with its subsequent amendments (Peru. Ministerio de Salud-DIGEMID, 1997; Peru. Law on Pharmaceutical Products, 2009; Peru. Ministerio de Salud- DIGEMID, 2011). According to this decree, the first category corresponds to natural resources for use in health, which are medicinal resources that have therapeutic properties based on traditional use on their label. The second category is natural products that are based on traditional uses and are presented in pharmaceutical form. The third category is herbal medicines, which are products presented in pharmaceutical form that have therapeutic activity and whose efficacy, safety, and quality have been scientifically demonstrated before the competent authority. The last category is homeopathic products, which comprise those products obtained in low doses of natural or chemical origin. All these products must have a sanitary registry.

As in the case of Brazil, in Peru, medicinal plants are partially included in the national health system through the Social Security Health System (EsSalud) as complementary medicine services at a national level. This includes natural pharmacies, which are those that dispense or prepare galenic preparations (tinctures, ointments, syrups, etc.) and monitor the efficacy, adverse effects, and interactions of medicinal plants that are used in the healthcare of patients of the Complementary Medicine Centers and Units of EsSalud (Peru, 2016; Palmer, 2019). These complementary medical services are in the process of being integrated into the services more broadly administered by the Ministry of Health and the Regional Governments (Vallejos-Gamboa et al., 2020). Conversely, the benefits of integrating traditional healing practices into formal medical clinics have been challenged as potentially leading to a diminishment of traditional healing knowledge due to growing reliance on medical clinics, in contrast to the integration of traditional knowledge in-home care and through local healers (Wolff, 2014).

## 4 Main initiatives for medicinal plant research in the Pan American countries

The research landscape in the Pan American countries is composed of specific initiatives and funding mechanisms that vary greatly from country to country and often involve collaborations between academic institutions and private industry. In many cases, both governmental and non-governmental agencies play vital roles in supporting medicinal plant research, thereby contributing to diverse initiatives that aim to explore the potential benefits of these natural resources to bridge the gap between traditional practices and modern scientific approaches. Across these countries, universities and individual research centers play a significant role in conducting medicinal plant research.

In the United States., research initiatives typically involve various organizations and agencies including the National Institutes of Health Office of Dietary Supplements (NIH-ODS) and National Center for Complementary and Integrative Health (NIH-NCCIH), U.S. Pharmacopeia (USP), American Herbal Pharmacopoeia (AHP), American Herbal Products Association (AHPA), and American Botanical Council (ABC), as well as several botanical research centers of excellence such as the National Center for Natural Products Research (NCNPR) of the University of Mississippi, that are partially funded by the FDA. These and other initiatives receive funding from different sources, including the government, such as FDA/NIH entities, among others, and often collaborate with private industry partners. In the United States., private research is also performed by companies that often do not publish the research due to its proprietary nature.

In Canada, medicinal plant research is multifaceted and involves universities, the natural health product industry, and government agencies. Funding comes from competitive grants awarded by the Natural Sciences and Engineering Research Council of Canada, the Canadian Institutes of Health Research, certain charitable foundations, and industry partnerships (Gray et al., 2013).

Regarding Pan American countries, many have government-sponsored initiatives that support medicinal plant research. These initiatives can include regulatory agencies, research institutes, and programs focused on integrating traditional medicine into their national health systems. For example, in Central America and the Caribbean, including the TRAMIL research group, international organizations, and development programs contribute to funding and collaboration in medicinal plant research. There is financial support from international entities such as WHO/PAHO, UNESCO, the EU, and other intergovernmental organizations (Alvarado-Guzmán et al., 2009). Also important for the natural product research activity in Pan Am countries has been the creation of the Italo-Latin American Society of Ethnomedicine (SILAE), established in 1993 (SILAE: Società Italo-Latinoamericana di Etnomedicina (Italo-Latin American Asian and African Society of Ethnomedicine, 2024). The fundamental objective of SILAE is to “promote the research, development, and use of medicinal and food plants in different countries of the world”. SILAE activities have supported doctorate, post-doctorate, and visiting scientist exchange programs, regular international conferences, and opportunities for collaborations between the different countries’ members.

In Mexico, COFEPRIS, the official entity that requests monographs on medicinal plants, does not provide financial support to research initiatives. The *Herbal Pharmacopeia of the United Mexican States* (FEUM: Farmacopea de los Estados Unidos Mexicanos, 2024) has financially supported the development of pharmacopeial monographs throughout the country. The most significant monographs of native plants have been provided by the Instituto Mexicano del Seguro Social, the National Federation of the Industry of Herbalism and Alternative Medicine, Traditional and Naturist (FNIHMATN), and academia, including public universities such as the Universidad Nacional Autónoma de México (UNAM), as well as private companies. Other academic institutions are also contributing to developing quality control procedures for the most widely used plants.

Medicinal plant research initiatives in Guatemala are conducted primarily by the academic sector, particularly the University of San Carlos, and are concentrated mainly on native plants and those introduced plants that are widely cultivated in the country. Research initiatives focus on ethnobotany, agrotechnology, scientific validation (pharmacology, phytochemistry), product formulations, and therapeutic use. Additionally, the Ministry of Public Health has a Commission for Traditional and Alternative Medicines that works to integrate some alternative health activities into the public health system.

Costa Rica is known for its historical efforts to protect its rich biodiversity (Tangley, 1986) with ethnobotanical and phytochemistry research focused on specific species (Arnaez et al., 2016), particularly from the University of Costa Rica (Navarro et al., 2015; Navarro et al., 2017; Navarro et al., 2019). A few studies have also been done on indigenous traditional uses, for instance from Cabecar, Guaymí (Hazlett, 1986), and the Bribrí groups (Pozo-García et al., 2020).

In Panama, much of the research on the medicinal use of plants is conducted by the Center for Pharmacognostic Research on Panamanian Flora (CIFLORPAN) at the University of Panama. There are also ecotours designed to study the medicinal plants of various indigenous tribes, including the Kuna, who are said to be among the most knowledgeable about medicinal plant use and who believe “plants were created and invested with spiritual powers to defend the life and soul of the people” (Lange, 2022).

In Colombia, there are several initiatives for medicinal plant research. Some of these initiatives are from the Humboldt Institute for Research on Biological Resources, the National University of Colombia, the Colombian National Herbarium, the National Institute of Natural Sciences, the Traditional Medicine Program from the Colombian government, and the Ministry of Science, Technology and Innovation (MINCIENCIAS). Each organization has funding mechanisms for the research and development of traditional medicines and works in various ways to integrate traditional medicine into the national health system.

In Peru, research institutes, universities, and government programs are active in medicinal plant research. Their funding comes from governmental sources and social health security initiatives. The most important organizations include The Research Institute of the Peruvian Amazon (IIAP), which is responsible for research on natural resources and Amazonian societies, and for the adoption of its results for the sustainable development of the Amazon. Also in Peru, the National Center for Intercultural Health (CENSI) conducts and promotes ethnographic, ethnobotanical, phytochemical, toxicological, pharmacological, and other related research on medicinal plants, according to the health needs of the population. Additionally, universities actively engage in programs to study traditional Peruvian healing practices (Donovan, 2013). The U.S. National Institutes of Health has funded anthropological research specifically aimed at reducing health disparities and addressing minority healthcare challenges.

Chile has several financing mechanisms through public funds (ANID, CORFO, Fondecyt, Fonis, FIA, FIC) and also private financing. And finally, in Argentina, public universities (Universidad de Buenos Aires), CONICET (National Science and Technology Council), and INTA (National Institute of Agricultural Technology) represent the primary initiatives for medicinal plant research (Niembro, 2020).

In Brazil, some initiatives are financially supported by governmental or non-governmental agencies. Through the National Program of Medicinal Plants and Phytotherapeutics of the Ministry of Health for the development of Live Pharmacies and making Phytotherapeutics available in the Unified Health System (health system supported by the governmental available to all Brazilians), specific Ministries projects such as the Environment and regional development to encourage the development of national production chains with social inclusion and through companies that develop Bioeconomy products, and non-governmental agencies, such as companies interested in development future herbal medicines.

## 5 Applicability of USP standards in the complex global regulatory landscape of medicinal plants in Pan American countries

USP standards are trusted in the United States and around the world. They are legally recognized in more than 40 countries and used in over 140 countries, in many cases through specific laws and regulations, and are helping to build stronger supply chains while fostering access to quality medicines, food ingredients, and dietary supplements (USP, 2024b). USP standards are developed with input from stakeholders in industry, academia, healthcare, and the government to ensure that the standards meet their needs, and can be used as an impartial, science-based resource for helping to ensure dietary supplement quality and ingredient integrity. USP standards are considered public standards because they are developed through a transparent process that includes an open public comment phase during which essential feedback is collected from stakeholders.

International organizations and regulators around the world recognize and trust the scientific rigor of USP’s standards, using them as the benchmark for quality. The use of USP standards helps support regulatory predictability in any country with USP standards integrated into their regulatory framework (Thakkar et al., 2020). For manufacturers, USP standards provide a common understanding across the supply chain of appropriate quality attributes for products and their ingredients. Meeting USP standards also helps allow for traceability and documentary evidence to support the quality of botanical articles throughout the supply chain. This, in turn, helps achieve sustainable sourcing, quality assurance, and minimizing risk for adulteration or fraudulent substitutions, particularly in cases of complex multiherbal preparations. Considering the complex global regulatory landscape of botanicals used as dietary supplements and herbal medicines in Pan American countries, the use of USP standards can be an expedient and cost-effective strategy for marketing herbal products globally (Sarma et al., 2023). Integrating USP standards into regulatory frameworks also supports in-country manufacturing by providing validated analytical methods for regulatory compliance. At the same time, in countries where USP standards are recognized, they reduce the need to perform additional analytical tests to meet different countries’ regulatory requirements.

A USP pharmacopeial standard is a combination of a documentary standard, such as a monograph or general chapter, and an associated physical reference standard (RS) (Ma et al., 2018). The specifications and analytical procedures used to define a specific article’s name and definition, including packaging and labeling requirements, are included in pharmacopeial monograph standards. Monographs provide tests, procedures, and acceptance criteria that help ensure the identity, strength, quality, and purity of an article. Thus, the core purpose of a monograph is to help ensure that products and their ingredients are of consistent quality.

Through the support of the BDSHM Expert Committee and the recommendations from several BDSHM Expert Panels (East Asia, South Asia, and Pan America), USP offers a portfolio of standards for the botanical dietary supplement and herbal medicine industry around the world (Dietary Supplements and Herbal Medicines; Dietary Supplements). The monographs are published in three different publications: *USP–NF*, the *Dietary Supplements Compendium* (*DSC*), and the *Herbal Medicines Compendium* (*HMC*):

• The *USP–NF* Dietary Supplements section (USP, 2024c) includes monographs for botanical articles regulated as dietary supplements in the United States as well as relevant general chapters, such as ⟨561⟩ *Articles of Botanical Origin*, ⟨563⟩ *Identification of Articles of Botanical Origin*, ⟨565⟩ *Botanical Extracts*, and ⟨203⟩ *High-Performance Thin-Layer Chromatography Procedure for Identification of Articles of Botanical Origin.*
• The *DSC* (USP, 2024d) includes excerpted standards from *USP–NF* and the *Food Chemicals Codex* (*FCC*) (USP, 2024g) that are relevant to the dietary supplement industry. Together with the standards are illustrations (chemical structures, chromatographic images, and botanical macroscopic and microscopic images) and Admission Evaluation documents. The latter are for introducing new monographs for dietary ingredients into the *USP–NF*; they provide safety assessment information based on typical intake levels, data from clinical studies and adverse events, toxicology data, potential interactions, and cautionary labeling statements, along with other valuable information.
• The *HMC* (USP, 2024e) includes standards for botanical articles used in traditional medicines. For inclusion into HMC, these articles need to be approved by a national authority for use as ingredients of herbal medicines or need to be included in a national pharmacopeia.

USP RS are a resource that industry can use to test for quality (USP, 2024f). They are highly characterized specimens of specific substances, including botanical dietary ingredients and herbal medicines (Sarma et al., 2023). USP RS are primary standards in jurisdictions that recognize them as such. When appropriate, they are calibrated relative to international reference materials such as those provided by the WHO. USP RS have official status when linked to tests in compendial documentary standards (monographs and general chapters) and are, therefore, generally accepted by regulatory authorities without additional qualification by users.

USP RS are compendial standards specified for use in conducting official *USP–NF* tests and assays. The RS candidate materials are supported by structural confirmation and are of the highest purity available. Their suitability for meeting applicable *USP–NF* monograph requirements is tested through multi-lab collaborative studies in ISO 17025-compliant labs. USP offers different types of botanical RS including powdered plant materials, characterized extracts, characterized fractions containing specific classes of compounds, and purified compounds suitable for a variety of compendial applications such as chromatographic fingerprinting, system suitability testing, purity testing, and limit testing.

## 6 Reflections on environmental sustainability and biodiversity conservation

Plants have been integral to human survival as foods, textiles, fuel, domiciles, and medicines throughout humanity’s history. For most cultures, plants, especially when employed medicinally and ceremonially, are not only a commodity to be used but are species that connect to our natural environment and, in some cases, contribute to our identity as a people. This is true today, whether it is the cultural large-scale propagation of tulips, apples, cannabis, and potatoes (Pollan, 2001) or the ancient Native American practices of controlled burning to encourage medicinal plant growth (Denis-Roy, 2015). It should be abundantly clear that humans are much more dependent on plants than plants are dependent on humans. The scientific discipline of ethnobotany encompasses this broad array of human-plant connections and chronicles the many ways that plants, especially medicinal plants, contribute to human and environmental health (Salmon, 2020; Schultes and von Reis, 2003).

The importance of medicinal plants in primary care has been underscored internationally by organizations such as the WHO, International Union for Conservation of Nature (IUCN), and World Wildlife Fund (WWF). Beginning in 1978, the Declaration of Ama Ata of these three organizations articulated that “health is a state of complete physical, mental, and social wellbeing, and not merely the absence of disease or infirmity.” The Declaration called on member nations to “protect and promote the health of all the people of the world” (WHO, 1978). This was followed by the Chiang Mai Declaration: Saving Plants That Save Lives, that specifically called on member nations “to recognize that medicinal plants are essential in primary healthcare, both in self-medication and in national health services” (Akerele et al. 1991).

The relevance of both Declarations was reaffirmed recently at the First WHO Traditional Medicine Global Summit convened by WHO/PAHO and hosted by the Ministry of Health Department of AYUSH in India (First Global Summit on Traditional). Many member nations embrace these Declarations and develop programs to realize the full integration of traditional and complementary medicine. During the summit, special emphasis was placed on the role that biological and cultural diversity plays as foreground to traditional knowledge and therapeutic pluralism in the creation of people- and community-centered health systems in the Americas. Because of the strong link that exists between biodiversity and traditional knowledge about health in the Americas, it is imperative to safeguard such knowledge and guarantee sustainability and biodiversity conservation amid climate change and other contemporary challenges to planetary health.

Similarly, in 2022, the Convention of Biological Diversity from the United Nation Environment Programme (United Nations, 2022), via *the Kunming-Montreal Global biodiversity framework- Draft decision submitted by the President,* also recognizes the important linkages between biological and cultural diversity, and “places biodiversity, its conservation, the sustainable use of its components and the fair and equitable sharing of the benefits arising out of the utilization of genetic resources, at the heart of the sustainable development agenda.”

USP has a strong commitment to environmental sustainability, as shown by its compliance with the relevant environmental laws and its efforts to continually improve conservation efforts. As a standard-setting body, USP has a specific role to play in the efforts to improve the herbal industry’s environmental footprint. When USP develops or revises a documentary standard, such as a product-specific monograph or a method guidance general chapter, the effect spans the entire supply chain and is multiplied through many users including suppliers, manufacturers, distributors, pharmacies, and healthcare providers. The USP BDSHM Expert Committee considers, IUCN Red List assessment and the Convention on International Trade in Endangered Species (CITES) during the process of admitting botanical monographs into the USP compendium.

To safeguard natural resources and send a warning about the status of some species, the third edition of the *Herbal Pharmacopeia of the United Mexican States*, published in 2021, introduces the Risk Category List in the Generalities section. The list includes the species named in the Official Mexican Standard NOM-059-SEMARNAT-2010, Environmental Protection; the species of wild flora and fauna native to Mexico; the risk categories and specifications for inclusion, exclusion, or change; and the list of species at risk (Estados Unidos Mexicanos - Secretaría de Medio Ambiente y Recursos Naturales, 2010). Similarly, most monographs of the American Herbal Pharmacopoeia include discussions regarding issues of sustainability of the monographed botanical, while the Canadian government similarly works in partnership with external partners, including indigenous peoples, to protect botanicals that may be threatened or endangered.

It is valuable to recognize that the use of herbal medicines did not decline in our modern era based on a scientific examination that found they did not work or were not safe, but rather due to how medical theories and therapeutic agents evolved. In recent decades, there has been an increasing worldwide interest in the potential benefits of herbal medicines, forcing nations to critically review the possible benefits of medicinal plants and to develop regulatory frameworks by which people needing or wishing to access herbal products may do so. It is relatively common knowledge that most modern medications were originally developed or conceived from natural products as represented by salicin from meadowsweet (*Spiraea ulmaria* L.) from which salicylic acid was obtained, which was later derivatized to acetylsalicylic acid and patented by Bayer in 1899 as Aspirin^®^, digoxin glycosides from foxglove (*Digitalis purpurea*), or vinca alkaloids from the Madagascar periwinkle (*Catharanthus roseus* (L.) G. Don). In addition to the contributions plants have made to modern drug development, most of the world’s population continues to rely on botanical-based products for their primary healthcare needs (Bodeker et al., 2005).

Overdevelopment and loss of habitat remain the primary threats to medicinal plants harvested in the wild. The same threats are similarly responsible for the loss of traditional cultures that carry with them the knowledge of medicinal plants. The consequences of this destruction are no more evident than throughout the Pan American region, with the rapid disappearance of huge swaths of Amazonian rain forests each year. While some efforts are being made to increase global attention to this environmental and cultural crisis, consumer demand is outpacing environmental sustainability. At the same time, lack of acknowledgment and inadequate research regarding the value of medicinal plants by national health authorities may be considered a threat to continued development of the traditional medicine knowledge base and conservation of medicinal plants. Following economic models for research on medicinal plants purely for the development of modern pharmaceuticals or commercial products, in general, detracts from a research focus aimed at promoting human health at the community level (Bussmann, 2013). In a review of medicinal plant research, it is reported that the majority of the most recent medicinal plant research has focused on the development of new drugs from plants, as opposed to the investigation of traditional plant use (Salmerón-Manzano et al., 2020). This results in a lack of allocation of resources for studying the traditional use of plants in the way they were historically utilized and minimizes the need for environmental stewardship.

In discussions about medicine and environmental health, it is not widely acknowledged that the use of herbal medicine, the promotion of sustainable wild-harvest practices, and the conservation of lands for medicinal plant cultivation represent a form of medicine that is highly environmentally sustainable and should be encouraged. Various countries throughout the world have plant and land conservation programs to greater or lesser degrees in the form of protected wilderness areas, ethnobotanical gardens, and training of traditional healers in conservation practices (WHO, 1993; Audet et al., 2012). Additionally, indigenous land management practices have been demonstrated to be sustainable in many areas of the world for generations. Although indigenous peoples represent only 5% of the population, they manage an estimated 20%–25% of the Earth’s land surface including areas that hold 80% of Earth’s biodiversity and approximately 40% of all terrestrial protected areas and ecologically intact landscapes. Recently, Brinckmann et al. (Brinckmann et al., 2022) performed a global estimation of medicinal and aromatic plant species in commercial cultivation including their conservation status. Of the identified commercial species, 82 species (2.5%) are threatened to some degree according to IUCN Red List categories and criteria. Additionally, 109 (3.4%) of the identified commercial species are included in CITES Appendices.

Lastly, there is great economic potential for medicinal plants as underscored in the Chiang Mai Declaration. Estimates project the world market for medicinal plants to reach $347 million (US$) by 2030 (Data Bridge Market Research, 2022). As the market for plants increases, efforts must be made to ensure their sustainability, so they are not simply approached from the perspective of pure commercial commodities. Sustainable harvesting and regenerative growth practices need to be integral to the development of herbal medicine quality control initiatives.

## 7 Final remarks

In addition to WHO recognizing that a large proportion of the world’s population relies on traditional medicines, including herbal medicines for their primary healthcare needs, consumers worldwide have demanded increased access to traditional healing modalities. Nations have responded in varied ways ranging from a complete embrace of WHO recommendations to tolerance and antagonism.

Countries throughout the Pan American region hold a tremendous amount of the planet’s natural resources within their borders and also maintain a rich heritage of cultural diversity, the identity of which is, in part, tied to indigenous plant knowledge. This includes the indigenous peoples of Canada and the US. At the same time, the desire for traditional herbal products is growing rapidly throughout the entire Pan American region, as it is throughout the rest of the world, as awareness of the benefits of herbal medicines increases.

Central to fulfilling the shared goal of health for all is the need for quality standards for all therapeutic agents used, whether they are conventional pharmaceuticals or traditional herbal medicines. The need to safeguard public health through the development of standards of identity, strength, composition, purity, and limits for contaminants is universal. The BDSHM Pan American Expert Panel’s current activities focus on developing a list of prioritized plants for standard development for USP-NF, DSC and HMC, depending on the regulatory status of the particular plant. The criteria for prioritization include, among others, cross-cultural use or being native across multiple Pan Am countries, evidence of therapeutic use, number of the products in the global market or commercialization, and use in national health systems. Expert Panel members are also anticipated to continue addressing technical, regulatory, policy and quality issues and advocate for the global safety of botanical dietary supplements and herbal medicines through scientific contributions, conferences, training and education in collaboration with several national and international organizations.

The U.S. Pharmacopeia can play a vital role in coordinating international efforts to establish quality standards for botanical ingredients used in dietary supplements and herbal medicines worldwide. The BDSHM Pan American Expert Panel, is in a unique position to help foster partnerships and information sharing by advancing the quality of herbal medicines and helping to improve global public health.
