# Supplementary material for: A U.S. Pharmacopeia (USP) overview of Pan American botanicals used in dietary supplements and herbal medicines
Source: Front Pharmacol. 2024 Aug 30;15:1426210. doi: 10.3389/fphar.2024.1426210 (PMC11392873; doi:10.3389/fphar.2024.1426210)
Supplement: Supplementary file 1 [file Table1.pdf]

Table 1. Historical timeline summarizing main milestones in the use of herbal medicines in the Pan Am region

Year/period	Milestone
Pre-colonization	Unrecorded use due to a predominantly oral history
Colonization:<br>1552 & 1574	*Libellus de Medicinalibus Indorum Herbis* (*Book of Medicinal Herbs of the Indies* also known as the *Badianus Manuscript*); *La historia medicinal de las cosas que se traen de nuestras Indias Occidentales (The medicinal history of the things that are brought from our West Indies)* by Spanish physician and botanist Nicholas Monardes (1574), translated by John Frampton (1596) as *Joyfull Newes out of the New-Found Worlde*
1587	In Brazil, the first description of the use of plants as medicine is made by Gabriel Soares de Souza.
Post- colonization:<br>1651	*Rerum Medicarum Novae Hispaniae thesaurus seu plantarum, animalium, mineralium, Mexicanorum historia* (*Inventory of medical items from New Spain, or, History of Mexican plants, animals and minerals*) published by Spanish ethnographer Francisco Hernández
1846	Publication of the first *Mexican Pharmacopoeia*
1882	Publication of the first *Chilean Pharmacopoeia* that is dominated by plant ingredients.
18th century	Cataloguing of Argentine herb use begins by Jesuits Pedro Montenegro and Segismundo Asperger
1900	In Argentina, Dr. Juan A. Dominguez establishes a museum (Museum of Pharmacobotany) containing plant and herbarium specimens creating a foundation for traditional herb use in the region (79).
1982	TRAMIL (Traditional Medicines in the Islands) initiative was established to help provide high-quality primary care to those who live within the Caribbean basin, leveraging the existing cultural use of medicinal plants, which is endemic throughout the region (32) and leading to the development of the *Caribbean Herbal Pharmacopoeia*. Approximately 90 plants approved throughout the region for use in national health care systems ((35, 36).
1994	Enactment of the Dietary Supplement Health and Education Act in the US that classifies herbal ingredients as a category of food and defines them as 'dietary supplements'.
1998	In the Ngawbere territory of Chile, a variety of traditional healers are formally trained as herbalists or health assistants to provide care to indigenous communities (96).
2000	Publication of the *Herbal Pharmacopeia of the United Mexican States*
1993-2016	Market surveys report approximately up to 150 herbs commonly sold in Mexican markets
2001-2023	In Mexico, it is reported that approximately 80–90% of the population utilizes some form of traditional herbal medicine and an estimated 50,000 shops sell herbal products; (18, 25, 26). 54% of health professionals and 49% of physicians reportedly used medicinal plants as an alternative therapy for several diseases
2002	The traditional Mapuche community of Chile assumes administration of the Maquehue Hospital and Health Centers of Boroa Filulawen and Ñi Lawentuwün in Araucanía Region to promote the use of traditional medical practices (94).
2004	Canada's Natural Health Products Regulations come into force, with guidance documents on standards for quality, safety and efficacy; and the first entries for their Compendium of Monographs - Natural Health Products are published
2006 & 2012	Approval of first two 'botanical drugs'; Veregen® (green tea sinecatechins) and Mytesi®, an extract prepared from latex of dragon's blood (*Croton lechleri*) (6).
2008	In Columbia, the *Colombian Vademecum of Medicinal Plants* containting 115 plant remedies is published (110, 111).
2015	Market vendors in Brazil report the medicinal use of 91 species of plants for 291 indications (108)
2009-2019	In Puerto Rico, medicinal plant use surveys record 118 plants as used in to treat a variety of conditions, Surveys of medicinal plant use conducted in Guatemala National

	with subsequent development of Vademecum of Medicinal Plants of Guatemala, and a Central American Medicinal Plants Vademecum that includes 150 medicinal plants that are traditionally used and commercially available in Central America. In Argentina, more than 1,600 plants recorded as used medicinally (68, 70, 71).
2022	USP launches the Pan American Botanical Dietary Supplements and Herbal Medicines (BDSHM) Expert Panel to support the development of quality control standards throughout Latin America. In Chile, a **native plant** medicines database, *Rizoma Database*, is launched and presents information regarding the use of 736 species of plants (97).
