# Supplementary material for: A U.S. Pharmacopeia (USP) overview of Pan American botanicals used in dietary supplements and herbal medicines
Source: Front Pharmacol. 2024 Aug 30;15:1426210. doi: 10.3389/fphar.2024.1426210 (PMC11392873; doi:10.3389/fphar.2024.1426210)
Supplement: Supplementary file 2 [file Table2.pdf]

Table 2. Representative Botanical Dietary Supplements and Herbal Medicines Regulations, Terminologies and Definitions, and Evidence of Integration into National Health Systems in Pam Am countries.

Country	Gov. Body/Regulator	Legal Document	Title/Description	Terminology/Definition
**Argentina**				
	Former Ministry of Health and Social Action/ ANMAT	Resolution 144/1998 Reformed by Resolution 1817/13	**Plant drugs, plant drug preparations, herbal medicines.** Include the import, export, preparation, fractionation, distribution - whether for consideration or free of charge - in national jurisdiction, or for inter-jurisdictional trade of plant drugs, preparations of plant drugs, herbal medicines, and the natural and legal persons involved in said activities	**Plant Drugs:** Whole plants or their parts, ground or powdered (flowers, fruits, seeds, tubers, bark, etc.), fresh or dried, as well as juices, resins, gums, latex, essential or fixed oils and other similar components, which are used pure or mixed in the preparation of phytotherapeutic medicines. **Phytotherapeutic Medicines:** Medicines defined following Article 1, subsection a) of Decree 150/92, but that do not meet the requirements established for the medicinal or pharmaceutical specialties defined in subsection d) of Article 1 of said regulation, and that contain as active ingredients pure plant drugs and/or defined mixtures of these and/or preparations of plant drugs, traditionally used for medicinal purposes, and do not contain chemically defined active substances or their mixtures even if they were isolated constituents of plants, except in cases so that they are justified.
		Disposition 2671/1999	**Phytotherapeutic medicines:** Standards for the authorization of establishments that produce, package/fraction, and importers of phytotherapeutic medicines	
		Disposition 2672/1999	**Phytotherapeutic products**: Guide to good manufacturing and control practices for phytotherapeutic products to guarantee the quality with which such products reach the market, manufactured, imported, and distributed	
		Disposition 4988/1999	**Dietary supplements:** Establishes registration conditions that owners of products authorized as dietary supplements should meet	
		Disposition 1788/2000	**Phytotherapeutic medicines - plant drugs**: List of plant drugs that must be excluded as constituents of phytotherapeutic medicines, because they have toxic effects for human beings, by themselves or by the method of administration	
		Disposition 2819/2004	Defines Good Manufacturing Practices for all medicinal products in the country, including phytotherapy products. Repealed by disposition 4159/2023	
		Disposition 2372/2008	Inspectors' guide on good manufacturing practices for medicinal products. It supersedes Disposition 2672/99	
		Disposition 5418/2015	Includes import, export, fractionation, elaboration, and trade of what the previous resolution defined as "phytotherapeutic medicine." It supersedes Disposition 2671/1999 and includes modifications in Disposition 2819/04 related to herbal medicines	The term **"Phytotherapeutic Medicine"** is replaced by **"Herbal Medicine,"** and **"Traditional Herbal Medicine"** is introduced to refer to medicinal plants that have been used for a long time in the country.
		Disposition 5482/2015	Habilitation requirements for import and export of herbal medicines establishments. It should comply with disposition 4159/2023	
	Health Quality	Joint Resolution 3/2020	Replaces Article 1381 of the Argentine Food Code with a	**Dietary Supplements**: are understood as products intended to

Country	Gov. Body/Regulator	Legal Document	Title/Description	Terminology/Definition
	Secretariat/ Secretariat of Food, Bioeconomy and Regional Development		new definition of Dietary Supplements	increase the usual dietary intake, supplementing the incorporation of nutrients and/or other ingredients in the diet of healthy people who, not being in pathological conditions, have basic dietary needs that are unsatisfied or greater than usual.<br>They must be administered orally and may be presented in solid forms (tablets, capsules, granules, powders, or others), liquid forms (drops, solution, or others), or other forms for gastrointestinal absorption.<br>They must contain, in simple or combined form: amino acids, proteins, lipids, carbohydrates, probiotics, vitamins, minerals, fibers and/or other ingredients with a nutritional or physiological role.<br>Herbs and/or vegetables.Herbs and vegetables contemplated in the Argentine Food Code should be used. They must comply with the specifications, requirements and limitations established regarding their content of substances limited in this Code, varieties and parts used.
Brazil				
	Ministry of Health/ ANVISA	Decree nº 5.813/ 2006<br>Reformed:<br>Decree nº 10.087 /2019	Approves the national policy on medicinal plants and herbal medicines and provides other measures	**Phytotherapeutic:** The product obtained exclusively from active vegetable raw material (comprises the medicinal plant, the vegetable drug, or the vegetable derivative), except isolated substances, with prophylactic, curative, or palliative purposes. It can be simple, when the active ingredient comes from a single medicinal plant species, or compounded, when the active ingredient comes from more than one medicinal plant species.
		National Policy for Medicinal Plants and Phytotherapeutics/2006	Establishes guidelines and priority lines for the development of actions by different partners around common objectives aimed at guaranteeing safe access and rational use of medicinal plants and herbal medicines, the development of technologies and innovations, as well as the strengthening of chains and production arrangements, the sustainable use of Brazilian biodiversity, and the development of the health	**Phytotherapy:** study of medicinal plants and their applications in promotion, protection, and recovery of health.<br>Medicinal plant. Plant species, whether cultivated or not, are administered by any means or form which exerts therapeutic action.<br>**Vegetable Derivative:** Product obtained from natural medicinal plants or plant drug, which contains substances responsible for therapeutic action and can be presented in the form of alcohol, wax, exudate, extract, fixed oil, volatile oil, dye and others.
		Disposition for the financing and execution standards of the Basic Component of Pharmaceutical Assistance within the scope of the Unified Health System	Establishes that the States, the Federal District, and the Municipalities are responsible for the selection, programming, acquisition, storage, stock control and expiration dates, distribution and dispensing of medicines and inputs of the Basic Component of Pharmaceutical Assistance, including medicinal plants, plant drugs, and plant derivatives for handling RENAME herbal medicine preparations in Live/Botanical Pharmacies and their compounding pharmacies	Evidence of integration into National Health System
		Resolution RDC nº 26/2014<br>Reformed:<br>RDC nº 66/2014<br>RDC nº 105/2016<br>RDC nº 106/2016<br>RDC nº 235/2018	Registration of Herbal Medicines and Registration and Notification of Traditional Herbal Products	

Country	Gov. Body/Regulator	Legal Document	Title/Description	Terminology/Definition
		RDC nº 317/2019<br>RDC nº 768/2022		
		Normative Instruction IN nº 4/2014	Guidance Guide for Registration of Herbal Medicines and Registration and Notification of Traditional Herbal Products	
		Normative Instruction IN nº 2/2014<br>Reformed:<br>IN nº 10/2014	Lists of Simplified Registration Herbal Medicines and Traditional Herbal Products	
		Resolution RDC nº 708/2022	Post-Registration Changes for Herbal Medicines and Traditional Herbal Products in Brazil	
		Resolution RDC nº 327/2019	Procedures for Granting Health Authorization for the Manufacture and Importation of Cannabis Products for Medicinal Use in Brazil	
		Resolution RDC nº 9/2015<br>Reformed:<br>RDC nº 205/2017<br>RDC nº 449/2020	Clinical Trial Regulations in Brazil	
		Normative Instruction IN nº 130/2022	Good Manufacturing Practices (GMP) for Herbal Medicines	
		Resolution RDC nº 406/2020<br>Reformed:<br>RDC nº 718/2022	Good Pharmacovigilance Practices for Marketing Authorization Holders of Human Medicinal Products	
**Canada**				
	Ministry of Health (Health Canada)	Food and Drugs Act Natural Health Products Regulations (SOR/2003-196)	These Regulations apply to:<br>(a) the sale of natural health products;<br>(b) the manufacture, packaging, labelling and importation for sale of natural health products;<br>(c) the distribution of natural health products; and<br>(d) the storage of natural health products for the purposes of any of the activities referred to in paragraphs (b) and (c).<br><br>For the purposes of these Regulations, a substance or combination of substances or a traditional medicine is not considered to be a natural health product if its sale, under the Food and Drug Regulations, is required to be pursuant to a prescription when it is sold other than in accordance with section C.01.043 of those Regulations.	**Natural Health Product:** a substance set out in Schedule 1:<br>(1) plant, algal, bacterial, fungal, or non-human animal material<br>(2) their extracts and isolates<br>(3) vitamins<br>(4) amino acids<br>(5) essential fatty acids<br>(6) a synthetic duplicate of a substance described in any of items (2) to (5)<br>(7) minerals, and<br>(8) probiotics;<br><br>Or a combination of substances in which all the medicinal ingredients are substances set out in Schedule 1, a homeopathic medicine or a traditional medicine, that is manufactured, sold or represented for use in<br>(a) the diagnosis, treatment, mitigation or prevention of a disease, disorder or abnormal physical state or its symptoms in humans;<br>(b) restoring or correcting organic functions in humans; or<br>(c) modifying organic functions in humans.<br><br>However, a natural health product does not include a substance set out in Schedule 2 (radiopharmaceuticals, biologic drugs, controlled drugs, drugs for injection, antibiotics, cannabis

Country	Gov. Body/Regulator	Legal Document	Title/Description	Terminology/Definition
				products with > 10 µg/g THC, isolated or concentrated phytocannabinoids and their synthetic duplicates), any combination of substances that includes a substance set out in Schedule 2 or a homeopathic medicine or a traditional medicine that is or includes a substance set out in Schedule 2.
		Regulations Amending the Food and Drug Regulations and the Cannabis Regulations (Supplemented Foods) (SOR/2022-169)	Amends existing regulations to add the category of Supplemented Foods	**Supplemental Ingredient:** Means a nutrient—including a vitamin, mineral nutrient or amino acid—or any other substance listed in column 1 of the List of Permitted Supplemental Ingredients and added as an ingredient to a food in accordance with the applicable conditions of use set out in columns 2 to 5.<br>**Supplemented Food:** Means a prepackaged product that belongs to a food category set out in column 1 of the List of Permitted Supplemented Food Categories and to which a supplemental ingredient has been added, but does not include:<br>(a) a food for special dietary use as defined in section B.24.001 and referred to in any of paragraphs B.24.003(1) f) to (f.2) and (h) to (j), even if the food for special dietary use is also a gluten-free food referred to in paragraph B.24.003(1)(g);<br>(b) a food that is labelled or advertised for consumption by<br>(i) infants as defined in section B.25.001,<br>(ii) children one year of age or older but less than four years of age, or<br>(iii) women who are pregnant or breastfeeding;<br>(c) any of the following foods set out in column I of the Table to section D.03.002:<br>(i) a food referred to in any of items 1, 2.1, 2.2, 4, 5, 7, 8, 9.1, 10 to 13, 15, 17 to 19, 21 to 25 and 27, and<br>(ii) prepackaged ice;<br>(d) a food that has not been processed or that has been minimally processed; or<br>(e) a beverage with an alcohol content of more than 0.5%;<br>Certain botanicals, which the industry notified Health Canada that they want to use as supplemented food ingredients, are subjected to a further safety assessment by Health Canada and may be added to the List of Permitted Supplemental Ingredients if found safe for use in supplemented foods within specified limits, e.g., green tea extract. Cannabis is explicitly excluded from supplemented foods; there are separate regulations covering edible cannabis products.
**Chile**				
	Ministry of Health, Subsecretary of Public Health/ISP	Supreme Decree No. 977/ 96		**Food Supplements:** Are those products made or specially prepared to supplement the diet for healthy purposes and contribute to maintain or protect characteristic physiological states such as adolescence, adulthood or old age. Its composition may correspond to a nutrient, a mixture of nutrients and other components naturally present in foods, including compounds such as vitamins, minerals, amino acids, lipids, dietary fiber, or their fractions. They may be sold in different forms of conventional release, such as such as powders, liquids, granules, dragees, tablets, tablets, capsules or others typical of medications.

Country	Gov. Body/Regulator	Legal Document	Title/Description	Terminology/Definition
				**Sport Foods:** Food products formulated to satisfy the requirements of healthy individuals, especially those who perform heavy and prolonged physical exercises. These foods will be composed of a food ingredient or mixture of these. One or more nutrients may be added, such as carbohydrates, proteins, vitamins, minerals and other components naturally present in foods, such as caffeine or those expressly authorized in this regulation. In its preparation, the standards of good manufacturing practices must be met.
		Supreme Decree No. 3/ 2010	Regulation of the national control system for pharmaceutical products for human use	**Phytopharmaceuticals:** Pharmaceutical specialties whose active ingredients come from the aerial or underground parts of plants or other plant materials are properly standardized.<br>**Traditional herbal medicines:** Products made up of plants or parts of plants, fresh or dried, whole or crushed, packaged and labeled by hand with the name used by popular custom in the field of Chilean cultural traditions.
**Central America**				
**Belize**<br>**Guatemala**<br>**Costa Rica**<br>**El Salvador**<br>**Guatemala**<br>**Honduras**<br>**Nicaragua**<br>**Panama**<br>**Dominican Republic**	Ministry of Health in each country	Central American Technical Regulations RTCA 11.03.64: 19	Products pharmaceuticals products natural medicinals for human use. Health registration requirements	**Extract:** Preparations of liquid consistency (fluid extracts and tinctures), semi-solid (soft extracts), or solid (dry extracts) obtained from natural drugs.<br>**Standardized extract:** extract that provides a minimum level or specific range of one or more constituents, whether or not they have pharmacological activity, provided that this one maintains the identity of the natural drug from which it comes.<br>**Natural preparation:** It is obtained from natural raw material through the fractionation process, solvent extraction, expression, distillation, purification, fermentation, concentration, or any other physical or biological process.<br>**Natural medicinal product:** product processed, industrialized, and labeled with medicinal properties, which contains in its formulation ingredients obtained from plants, animals, minerals, or mixtures of these. They may contain excipients in addition to natural material. Natural medicinal products to which substances are added, chemically synthesized, or isolated from natural material as responsible for the pharmacological activity are not considered natural medicinal products.<br>**Traditional medicinal natural product:** It is one whose use and safety of natural active substances are justified by ethnomedicinal reports, technical and scientific documentation, indexed publications, or documents endorsed by a committee of experts. It is used by oral, topical, or other route that does not require sterility.
		Central American Technical Regulations RTCA 03.11.56: 09	Quality Verification	
		Central American Technical Regulations RTCA 11.04.41: 06	Labeling Requirements	
		Government of the	Includes information on 40 medicinal plants used for	Evidence of integration into National Health System

Country	Gov. Body/Regulator	Legal Document	Title/Description	Terminology/Definition
		Republic of Guatemala. Ministry of Public Health and Social Assistance. Comprehensive Health Care Standards for First and Second Level//2018	primary health care	
		Guatemalan Network of Integrative Medicine against Covid (Regmic). Natural Formulary for COVID-19	Includes general information on natural medicinal products, their correct use from the point of view of their origin, ways of extracting active substances (especially from medicinal plants), types of finished products, as well as examples of medicinal plants. It also contains a series of simple preparations, for primary care at home, of the symptoms associated with COVID-19	Evidence of integration into National Health System
**Colombia**				
	Ministry of Health and Social Protection / INVIMA	Resolution 2834/2008	Establishes the adoption of the Colombian Vademecum of Medicinal Plants and the guidelines for its update	**Phytotherapeutic product.** It is a packaged and labeled medicinal product whose active substances come from medicinal plant material or their combination; they are presented in pharmaceutical form and used for therapeutic purposes. They can also come from extracts, tinctures, or oils. Their formulation may not contain isolated and chemically defined active ingredients. Products obtained from medicinal plant material that have been processed and obtained in pure form will not be classified as phytotherapeutic products.
		Resolution 5107/2005	Establishes the Good Manufacturing Practices to manufacture phytotherapeutic products	
		Decree 2266/2004	Labeling Requirements	
		Decree 1156/2018	Regulates the commercialization registration procedure for phytotherapeutic products, quality control drug product, sanitary vigilance and other provisions	Evidence of integration into National Health System
**Mexico**				
	Health Secretary/ COFEPRIS	Health General Law/1984 Reformed: 2024	Establishes the bases and modalities for access to health services	**Food Supplements.** Herbal products, plant extracts, traditional foods, dehydrated or fruit concentrates, whether or not added, vitamins or minerals, which can be presented in pharmaceutical form and whose purpose of use is to increase total dietary intake, complement it or replace any of its components.<br>**Herbal Product.** Products made with plant material or some derivative thereof, whose main ingredient is the aerial or underground part of a plant or extracts and tinctures, as well as juices, resins, fatty and essential oils, presented in pharmaceutical form, whose therapeutic efficacy and safety have been scientifically confirmed in national or international literature.
		Regulation of Health Products / 1998 Reformed: 2021	The purpose of this regulation is to regulate the health control of supplies and herbal remedies, as well as the establishments, activities, and services related to them	**Herbal Remedies**. Are prepared from medicinal plants, or their parts, individual or combined, and their derivatives, presented in pharmaceutical form, to which popular or traditional knowledge is attributed relief for some participating or isolated symptoms of a disease.<br>**Herbal Medicines**. In addition to containing plant material, they may add excipients and additives to their formulation.
		2018 Edition of the Basic Table of Herbal	Establishes that the public institutions of the National Health System should only use the supplies, including	Evidence of integration into National Health System

Country	Gov. Body/Regulator	Legal Document	Title/Description	Terminology/Definition
		Medicines.	herbal medicines, established in the basic table for the first level of medical care and for the second and third levels, the catalog of supplies.In this edition, five new herbal medicines (added to 17 existing) to the Basic Table of Herbal Medicines, which may be used at the three levels of care in the adult population	
		2020 Edition of the Book of Herbal Medicines of the National Compendium of Health Supplies	The General Health Council, the Secretary of National Defense, the Secretary of the Navy, the Secretary of Health, the Mexican Institute of Social Security, the Institute of Security and Social Services of State Workers and the Health Services of Petróleos Mexicanos, to analyze appropriate updates to the National Compendium of Health Supplies, in order to consider the inclusion of various herbal medicines	Evidence of integration into National Health System
**Peru**				
	Health Ministry/ DIGEMID	Law N.° 29459	Law on pharmaceutical products, medical devices and health products	Classifies pharmaceuticals products into:<br>a) Medicines<br>b) Herbal Medicines<br>c) Dietetic product and sweeteners<br>d) Biological Products<br>c) Galenic product<br>**Herbal medicines**. Medicinal products in pharmaceutical dosage form that contain herbal preparations have been scientifically demonstrated to be efficacious, safe, and of high quality.<br>**Dietetic product**. That product whose purpose is to complement the normal diet that consists of concentrated sources of nutrients or other substances that have a nutritional or physiological effect, in simple or combined and dosed form. They are only used orally.
		Supreme Decree Nº 016- 2011-SA	Regulations for Registration, Control and Surveillance Health of Pharmaceutical Products, Medical Devices and Health Products<br><br>Establishes that herbal medicines and natural products for health use require sanitary registration; and that medicinal plants and natural resources of mineral origin do not require health registration, if in their label they don't indicate therapeutic properties	
		Supreme Decree Nº 010- 97-SA	Regulations for the Registration, Control and Health Surveillance of Pharmaceutical and Related Products	**Natural Resource of Use in Health.** Resources of nature (plant, animal, or mineral origin) that haven't been processed. They have been fragmented, dehydrated, or pulverized and are used in producing herbal medicines. If on their label they don't indicate therapeutic properties, they don't need sanitary registration. (Art. 8º D.S. Nº 004-2000-SA).<br>**Natural Products of Use in Health.** Simple or complex industrial preparations are based on one or more natural resources, using the isolated or synergistic properties of these resources, which are traditionally used by populations of one or more national and international cultures. (Art. 9º D.S. Nº 004-

Country	Gov. Body/Regulator	Legal Document	Title/Description	Terminology/Definition
				2000-SA).
		General Management Resolution Nº 842 - GG- ESSALUD- 2016	Product Request Resources and Complementary Medicine Supplies	Evidence of integration into National Health System<br>**Herbal Medicine for Medicinal Use**. Medicinal products made with herbal preparations, presented in pharmaceutical form, have therapeutic activity, and have been scientifically demonstrated to be effective, safe, and of good quality by the competent authority. Requires Health Registration.<br>**Herbal preparations.** Herbal preparations are the basis of finished products and may consist of crushed or powdered herbal materials, extracts, tinctures, and fatty oils from herbal materials. They are produced by extraction, fractionation, purification, concentration, and other biological or physical processes.<br>**Medicinal plant**. A wild or cultivated plant that is used as a remedy to prevent, alleviate, cure, or modify a normal or pathological physiological process, that is, for medicinal purposes; as a source of drugs or their precursor.
**United States**				
	Department of Health and Human Services/FDA	Public Law 103-417/1994	Dietary Supplement Health and Education Act of 1994 establishes definitions:<br>• Safety of dietary supplements and burden of proof on FDA to prove unsafe.<br>• Dietary supplement claims (pre-notification requirements)<br>• Statements of nutritional support<br>• Dietary supplement ingredient labeling and nutrition information labeling<br>• New dietary ingredient requirements<br>• Good manufacturing practices<br>• Facility registration requirements	The term **Dietary Supplement**:<br>(1) means a product (other than tobacco) intended to supplement the diet that bears or contains one or more of the following dietary ingredients:<br>(A) a vitamin;<br>(B) a mineral;<br>(C) an herb or other botanical;<br>(D) an amino acid;<br>(E) a dietary substance for use by man to supplement the diet by increasing the total dietary intake or<br>(F) a concentrate, metabolite, constituent, extract, or combination of any ingredient described in clause (A), (B).
		Code of Federal Regulations Part 111	Current good manufacturing practice in manufacturing, packaging, labeling, or holding operations for dietary supplements<br>Quality means that the dietary supplement consistently meets the established specifications for identity, purity, strength, and composition, and limits on contaminants	
		H.R.3448 - Public Health Security and Bioterrorism Preparedness and Response Act of 2002	Facilities that manufacture, process, pack, or hold dietary supplements or dietary ingredients for consumption in the United States must register with the FDA before beginning such operations, as required by the Public Health Security and Bioterrorism Preparedness and Response Act of 2002 and implementing regulations	
		Public Law No: 109-462/2006	S.3546 - Dietary Supplement and Nonprescription Drug Consumer Protection Act<br>Considers a serious adverse event report to be: (1) a safety report that may be accompanied by a statement, which shall be included in any public disclosure of the report, that denies that the report or the records constitute an admission that the product involved caused or contributed to the adverse event; and (2) a record about an individual and a medical or similar file the disclosure of which violates the	

Country	Gov. Body/Regulator	Legal Document	Title/Description	Terminology/Definition
			Freedom of Information Act unless all personally identifiable information is redacted.	
		Botanical Drug Development: Guidance for Industry/ 2006	Pharmaceutical Quality<br>Chemistry, Manufacturing, and Controls<br>Submission guidelines for botanical drugs to be submitted in new drug applications (NDAs) and specific recommendations on submitting investigational new drug applications (INDs) in support of future NDA submissions for botanical drugs. In addition, this guidance provides general information on the over-the-counter (OTC) drug monograph system for botanical drugs	A botanical product may be classified as a food (including a dietary supplement), drug (including a biological drug), medical device, or cosmetic under the Federal Food, Drug, and Cosmetic Act (FD&C Act). Whether an article is a food, drug, medical device, or cosmetic depends in large part on its intended use, though for some product types, other factors must also be considered. Intended use is established by, among other things, the product's labeling, advertising, and the circumstances surrounding its distribution. A botanical product intended for use in diagnosing, curing, mitigating, or treating disease would meet the definition of a drug under section 201(g)(1)(B) of the FD&C Act and would be subject to regulation as such. A botanical product intended to prevent disease would also generally meet the definition of a drug under section 201(g)(1)(B) and be regulated as a drug.

ANMAT (National Administration of Drugs, Foods and Technologies); ANVISA (Brazilian Health Regulatory Agency); COFREPRIS (Federal Commission for the Protection from Sanitary Risk); DIGEMID (General Directorate of Medicines, Supplies and Drugs); FDA (Food and Drug Administration); ISP (Institute of Public Health); INVIMA (National Institute for Food and Drug Surveillance); RENAME (National List of Essential Medicines).
